# Druggability of Sodium Calcium Exchanger (NCX): Challenges and Recent Development

**DOI:** 10.3390/ijms26188888

**Published:** 2025-09-12

**Authors:** Antonia Scognamiglio, Angela Corvino, Giuseppe Caliendo, Ferdinando Fiorino, Elisa Perissutti, Vincenzo Santagada, Beatrice Severino

**Affiliations:** Department of Pharmacy, School of Medicine, University of Naples Federico II, Via D. Montesano 49, 80131 Naples, Italy; antonia.scognamiglio@unina.it (A.S.); angela.corvino@unina.it (A.C.); caliendo@unina.it (G.C.); fefiorin@unina.it (F.F.); perissutt@unina.it (E.P.); santagad@unina.it (V.S.)

**Keywords:** sodium calcium exchanger (NCX), inhibitors, activators, drug design, neurodegeneration

## Abstract

Na^+^/Ca^2+^ exchangers (NCXs) are membrane transporters crucial for calcium homeostasis in excitable tissues, particularly in the central nervous system. Growing evidence indicates that NCX dysfunction contributes to calcium overload and neuronal damage in several neurological conditions. Thus, pharmacological modulation of NCX isoforms (NCX1, NCX2, and NCX3) has emerged as a potential therapeutic strategy for disorders such as stroke, multiple sclerosis (MS), amyotrophic lateral sclerosis (ALS), Alzheimer’s disease (AD), and Parkinson’s disease (PD). However, the identification of selective modulators directed at specific NCX isoforms, or even different splice variants, remains challenging and limits their clinical validation. This Review aims to provide an updated overview of small-molecule NCX modulators, described over the last two decades. Chemical structures, mechanisms of action, and isoform specificity are discussed, along with the most commonly used biological assays for their functional evaluation.

## 1. Introduction

**Figure 1:**
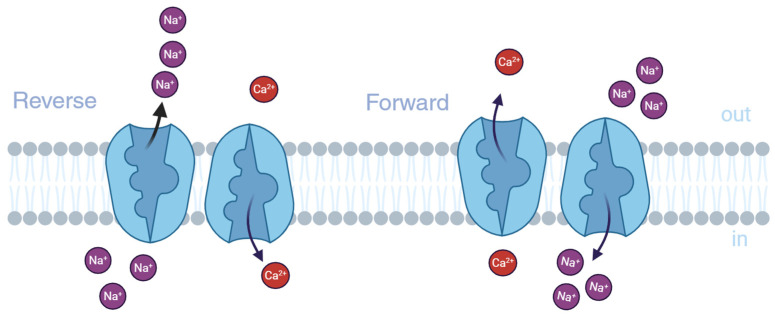
Simplified representation of the ion transport modes by the Na^+^/Ca^2+^ exchanger created in https://BioRender.com (accessed on 5 February 2025). In the forward mode (**right**), one Ca^2+^ ion is extruded from the cytosol to the extracellular (EC) environment through the plasma membrane, followed by the entry of three Na^+^. The opposite transport occurs when the NCX is operating in the reverse mode (**left**).

The physiological levels of calcium (Ca^2+^) and sodium ions (Na^+^) are controlled by various Ca^2+^-handling proteins, selective ion channels, and ATP-dependent pumps. Among these, the Na^+^/Ca^2+^ exchanger (NCX) is a key transmembrane protein that facilitates the bidirectional exchange of Na^+^ and Ca^2+^ across the plasma membrane. Typically, NCX operates with a stoichiometry of three Na^+^ ions exchanged for one Ca^2+^ ion, primarily extruding Ca^2+^ from the cytoplasm to maintain lower intracellular concentrations compared to extracellular levels. This forward mode (Ca^2+^ extrusion) is driven by the Na^+^ electrochemical gradient established by the Na^+^ pump. However, under certain conditions, such as increased intracellular Na^+^ or membrane depolarization, NCX can reverse its function, allowing Ca^2+^ influx while extruding Na^+^ (Figure 1). The significance of NCX in humans varies across different tissues; it plays a dominant role in regulating physiological responses in cardiac muscle, smooth muscle, renal function, and the central nervous system (CNS). In the CNS, dysregulation of NCX has been implicated in several neurodegenerative diseases and conditions such as Alzheimer’s disease, Parkinson’s disease, and post-ischemic brain injury [1,2,3,4]. Altered NCX activity can lead to disrupted calcium homeostasis, contributing to neuronal excitotoxicity and cell death.

Recent studies have highlighted that NCX isoforms (NCX1–3), while generally low in abundance, are critical for rapid Ca^2+^ fluxes suited to specific cellular demands. The expression levels of these isoforms are linked to various diseases, suggesting that targeting tissue-specific NCX variants could offer therapeutic potential. In this regard, structural studies have revealed important regulatory mechanisms of NCX, elucidating a common module for decoding Ca^2+^-induced allosteric signals in eukaryotic variants. However, challenges remain in fully characterizing the structural differences among isoforms [5].

The numerous efforts to understand the intricate mechanisms governing NCX’s function have shed light on its role in health. They pave the way for potential therapeutic strategies to restore regular ion transport in diseases characterized by ionic imbalance. This targeted approach might ultimately lead to innovative treatments that address the underlying causes of neurodegeneration or at least merely alleviate symptoms. Yet, despite two decades of research, the landscape of potential clinical applications remains fragmented. In this review, we showcase the chemical diversity of NCX modulators reported in the literature, their mechanisms of action, selectivity towards NCX1, NCX2, and NCX3 isoforms, and consider the biological profile and significant obstacles that restrict their translation into clinical use. We consider data from different experimental contexts, including human post-mortem tissue, mammalian in vivo models, and in vitro cellular systems. While species-specific differences in NCX isoform regulation exist, we have explicitly indicated the experimental context (human, animal model, or cellular system) for each study discussed. Findings are examined from a cross-species perspective to provide a comprehensive overview of NCX isoform regulation.

## 2. Functions and Pathogenetic Role of NCX Isoforms

### 2.1. NCX Genes and Splice Variants

The mammalian Na^+^/Ca^2+^ exchange proteins are classified into three isoforms, known as NCX1, NCX2, and NCX3, which are encoded by the genes *SLC8A1*, *SLC8A2*, and *SLC8A3*, respectively. These isoforms and their splice variants exhibit tissue-specific expression and display distinct regulatory features, while sharing approximately 70% sequence identity. NCX2 displays a 65% sequence identity with NCX1, whereas NCX3 shares a 73% sequence homology with NCX1 and 75% with NCX2 [6].

NCX1 is ubiquitously expressed in all mammalian cells, with at least 17 distinct splice variants created through alternative splicing. They are widely expressed in both excitable (such as cardiomyocytes and neurons) and non-excitable tissues (e.g., pancreas and kidney), and among them, the cardiac NCX1.1 variant is the most extensively investigated due to its critical involvement in heart contractile activity.

NCX2, mainly expressed in the brain and spinal cord, may also be found in the kidney and gastrointestinal tissues, even though no splicing variants have been observed for this isoform. NCX3 is primarily found in the brain and skeletal muscle, where at least five splice variants have been identified. These variants are involved in stress conditions such as neuronal excitotoxicity and stroke, as well as in memory formation through their implication in long-term potentiation in the hippocampus. Additionally, their presence in bone tissue and the immune system suggests a role in bone formation and hormone secretion [7,8].

The numerous variants originate from the combinations of six small exons (A, B, C, D, E, and F) in the splicing segment located in the F-G loop of one of the two Ca^2+^-binding domains, CBD2. Exons A and B are mutually exclusive, with exon A predominantly expressed in excitable tissues, like the heart and brain, and exon B in non-excitable tissues, like the kidney, stomach, and intestine. Their combination with varying compositions of “cassette” exons gives rise to the distinct NCX variants, which affect response kinetics, dynamic range, and allosteric sensor affinity, leading to diverse responses to regulatory Ca^2+^.

Specifically, Ca^2+^ activates the brain (AD), cardiac (ACDEF), and kidney (BD) variants, though Ca^2+^-induced relief from Na^+^-dependent inactivation is observed only in the cardiac and brain variants. The stoichiometry of Ca^2+^ binding to CBD2 varies from 0 to 3 by inserting specific residues at three distinct positions, thereby justifying the substantial differences in terms of kinetics and activity modulation among all the isoforms and splice variants.

### 2.2. Regulation of NCX Isoforms and Their Splice Variants

The three mammalian isoforms (NCX1-3) and their splice variants are expressed tissue-specifically to match cells’ demands under ever-changing physiological conditions.

As a secondary-active transport mechanism, NCX operates in either the forward or reverse mode, depending on the electrochemical driving force of the membrane potential and the ionic gradient across the membrane. Compared to PMCA and SERCA, which are high-affinity/low-capacity ATP-dependent pumps tightly regulated by calmodulin and reversible phosphorylation to ensure fine control of Ca^2+^ at low concentrations, NCX activity is primarily governed by the transmembrane gradients of Na^+^ and Ca^2+^ and by allosteric activation through intracellular Ca^2+^ binding. This distinctive mode of regulation reflects its role as a rapid, high-capacity safety valve during Ca^2+^ overload, in contrast to the fine-tuning functions of PMCA and SERCA. These unique features highlight why NCX deserves special consideration as a therapeutic target in conditions characterized by disrupted calcium homeostasis [4,9].

The exchange rates are also under the control of regulatory processes that integrate a wide range of feedback and feedforward signals.

The allosteric regulation of eukaryotic NCXs occurs in the cytoplasm, which is also the target of several recently discovered inhibitory drugs. In contrast to prokaryotic NCX, which contains a short loop between TM5 and TM6 (5L6) of approximately 16–32 residues, eukaryotic NCXs exhibit a large cytosolic 5L6 loop (approximately 520 residues), which is the site of multiple controlling mechanisms.

The dynamic regulation of Na^+^/Ca^2+^ exchanger relies on diverse structural elements:

- The auto-inhibitory sequence (XIP);
- A two-helix bundle (THB) module;
- Two regulatory Ca^2+^ binding domains 1 (CBD1) and 2 (CBD2);
- A short palmitoylation helix (TMH2).

**Figure 2:**
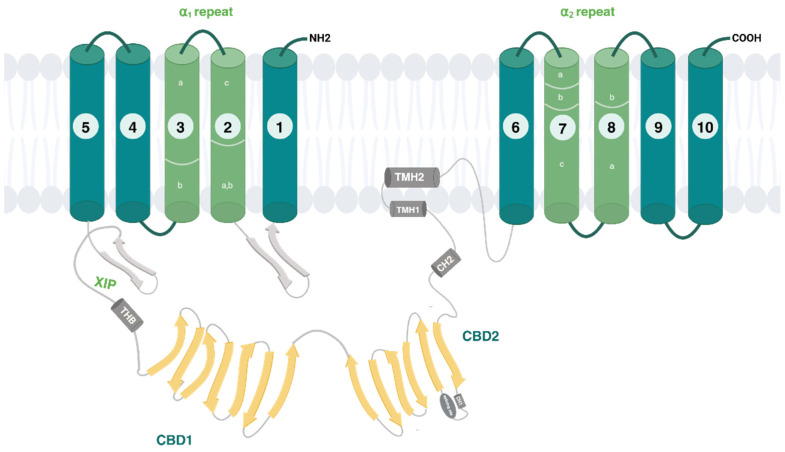
The overall structure of human cardiac NCX1 created in https://BioRender.com (accessed on 15 March 2025). (adapted from [13] https://doi.org/10.1038/s41467-023-41885-4).

Cytosolic Na^+^ and Ca^2+^ indirectly control the activity of the NCX proteins, exerting what is known as ion-dependent regulation [10]. An increase in cytosolic Ca^2+^ activates all NCX isoforms through the binding to the high-affinity sites of CBD1. Conversely, intracellular Na^+^ rise reduces the transport activity by promoting the exchanger’s transition into a Na^+^-dependent inactivated state [11]. When Na^+^ concentrations inside the cell rise, the ion binds to the transport site of the antiporter, initially triggering a rapid outward Na^+^/Ca^2+^ exchange current, which is subsequently followed by inactivation. Specifically, Na^+^-dependent inactivation proceeds following the bonding of three Na^+^ ions to the inward-facing exchanger. Even if the identity of the Na^+^-inactivation site is not well established, CBDs are not involved [12]. Structural analysis of human cardiac NCX1 has shown that, in the inward-facing conformation, the β-hairpin located between TMs 1 and 2ab may interact with the XIP region to form a β-hub (Figure 2). This β-hub can rapidly interact with the cytosolic domain through the CH2 helix of CBD2, resulting in a stable inactivated complex. This complex effectively locks TMs 1 and 6 in the inward-facing state, thereby hindering their motion and preventing ion translocation [13].

The two CBDs stand in the large portion of the cytosolic domain, adopting an elongated and broad V-shape with a hinge angle of 120°. CBD1, in a distal position relative to the TM domain, extends its N-terminus deeply into the cytosol, linking the XIP domain via a long and disordered loop. In contrast, CBD2 is located closer to the TM domain, with its C-terminus segment directly interacting with the transmembrane domains. This interdomain contact is the essential portion for NCX inactivation. For instance, the Ca^2+^ binding to CBD2 causes the β-hub disassembly and TMs’ release, leaving them free for the exchange architecture. Even under nominally Ca^2+^-free conditions, CBD1 remains in a Ca^2+^-bound state, with all four binding sites occupied, reflecting its high affinity for Ca^2+^. These Ca^2+^ ions are grouped near the interface of CBD1 and CBD2, stabilizing the local structure and promoting interactions between the two domains. Since the four Ca^2+^-binding sites on CBD1 are far from the transmembrane region, they do not directly influence its ion exchange function. Substantially, the Ca^2+^ orchestrates the transport machinery, tethering the CBDs and restricting their interdomain movements to communicate the allosteric message to membrane-associated TM segments, as observed in the most recent work on the full-size mammalian NCX1.4 variant [14]. This study proposes the Ca^2+^-induced rigidification of CBD mobility as a shared regulatory mechanism among mammalian NCX proteins. Additionally, the dynamic features of interdomain movements may be further influenced by differences in exon composition across various isoforms or splice variants [14]. In this regard, the CBD2 domain serves as a splicing site, thereby predetermining a specific ability of a given NCX variant to exert Ca^2+^-dependent alleviation of Na^+^-induced inhibition. This construction aligns with the functional studies on the full-size mammalian NCX variants, highlighting the differences between the cardiac NCX 1.1 and brain NCX 1.4, as well as NCX1.3, NCX3.1, and NCX2. In the latter variants, predominantly expressed in the kidney, skeletal muscle, brain, osteoclasts, and osteoblasts, the mechanism of Ca^2+^-dependent alleviation of Na^+^-inactivation is lacking [14,15,16,17].

Besides the Ca^2+^ binding to CBD2, PIP2 interaction with a putative site near CBD2 could alleviate Na^+^-induced inactivation. The auto-inhibitory mechanism relies on a polybasic region called “XIP domain”, located in the juxtamembrane area at the beginning of the 5L6 loop (residues 219–238), which establishes extensive interactions with the intracellular component of transmembrane helices. The PIP2 binding to this portion was proposed as an activating mechanism of NCX1.1 as well; in fact, the Na^+^-dependent release of PIP2 from this region suppresses the NCX activity, probably exposing the binding site for the interaction with a distal region in the N-terminal portion of the f-loop (P1 domain, 562–688aa) [18,19]. Hence, Na^+^ and PIP_2_, as negative and positive modulators, may govern the switch from the steady-state equilibrium to the active or inactive states of NCXs. Alongside allosteric modulation, NCX isoforms undergo post-translational modifications, such as S-palmitoylation, that establish local conformational changes within the intracellular loop, thereby affecting the shape rearrangements that promote ion occlusion and release. The XIP domain physically interacts with a segment of twenty amino acids (709–728), and this interaction is very close to the TMH2 domain (residues 739–756), which contains a palmitoylatable cysteine (Cys-739) and an amphipathic α-helix [20]. Owing to the proximity of these sites, it has been proposed that palmitoylation may remodel this region of the protein controlling XIP binding and NCX inactivation [21,22]. Although most of the studies are performed on the NCX1 isoform, the presence of Cys739 in the NCX3 isoform suggests that it may undergo palmitoylation, even if specific studies are still needed. Structural divergence among NCX orthologs constitutes the fundamental basis for the regulatory heterogeneity observed in eukaryotic NCX isoforms, enabling each isoform/splice variant to meet tissue-specific demands. Advancements in elucidating the structure-based fundamentals of allosteric regulation, particularly those involved in Na^+^-dependent inactivation characteristic of NCX1 and NCX3, could pave the way for the rational development of isoform-selective modulators with potential biomedical significance.

### 2.3. NCX Isoforms in Neurological Diseases

In recent decades, research has broadened our understanding of NCXs, showing that their functions at the plasma membrane go beyond regulating cytosolic Ca^2+^ levels. Physiologically, NCXs play a role in modulating cellular excitability, supporting Na^+^-dependent transporters, regulating ion channel activity, and enhancing endocytic responses during stress. Moreover, evidence suggests that NCX antiporters are also located in cellular compartments, such as the mitochondria and the nucleus [23,24]. The widespread distribution highlights the crucial role of NCX in maintaining cellular energy balance, promoting growth, and ensuring survival.

The connection between Ca^2+^ signaling and brain functions clarifies the Na^+^/Ca^2+^ exchanger’s potential role in neurodegenerative diseases, marked by the gradual and selective loss of neuronal systems. Despite their diverse clinical presentations, these disorders share common molecular pathways concerning Ca^2+^ homeostasis and signaling disruption. Key factors include impaired Ca^2+^ buffering capacity, dysfunctional Ca^2+^ channel activity, and alterations in other Ca^2+^-regulating proteins, often driven by excitotoxicity, disturbed energy metabolism, and oxidative stress. Abnormal intracellular Ca^2+^ accumulation leads to neuronal cell death by inducing proteases and caspase activity, as well as triggering destructive catabolic processes involving lipases and nucleases.

Numerous studies have investigated this aspect, analyzing the mechanistic basis related to the onset and progression of neurological and neurodegenerative conditions; the most relevant findings are summarized in Table 1. These studies indicate that balancing NCX operation is essential to handle cellular homeostasis in these neurological disorders, whether they occur acutely, as in the case of stroke, or chronically, as in Parkinson’s and Alzheimer’s diseases. However, the clinical potential of employing NCX is still under examination.

#### 2.3.1. Cerebral Ischemia—Inadequate Blood Flow to the Brain

Caused by either physical obstruction (ischemic stroke) or blood vessel (hemorrhagic stroke), leads to dramatic oxygen deficiency and loss of essential nutrients needed for brain cell survival. Depending on the severity and length of the ischemic episode, the extent of brain damage depends on the timeliness of diagnostic and intervention.

The rapid cell death triggered in the ischemic core region makes its recovery impracticable; on the other hand, the surrounding region, known as the penumbra, might be restored by taking advantage of the short therapeutic window. In this area, multiple damaging mechanisms are brought about by dysregulation of intracellular ionic balance and ROS production. The progressive increase in [Na^+^]_i_, originating from the Na^+^-K^+^ ATPase blockade, designates the onset of an anoxic insult responsible for cell swelling and microtubular disruption, ultimately resulting in cell necrosis. At this stage, NCX is forced to operate in reverse mode to ensure neuroprotection by reducing Na^+^ overload, thus, in the meantime, raising the [Ca^2+^]_i_ through the passageway across the membrane. This working mode promotes ER stores’ refilling, which are depleted due to anoxia followed by reoxygenation, and in turn delays the ER stress in neurons. In the later phase of neuronal anoxia, Ca^2+^_i_ overload takes place. NCX supports its extrusion via the forward mode of operation and protects neurons from Ca^2+^ overload-induced neurotoxicity and cell death.

The three NCX isoforms are expressed in the brain regions with different distribution patterns [25], and commonly in the same neurons; nevertheless, these proteins could be differently involved in Ca^2+^ handling during the hypoxia-anoxia insult. Notably, in vitro studies pointed out that NCX3 is barely affected by the inhibitory mechanism caused by ATP depletion, significantly contributing to the maintenance of calcium homeostasis under experimental conditions mimicking ischemia. The assumption has been proved by Secondo and colleagues, who observed that BHK cells singly transfected with NCX3 exhibited an intrinsic resistance to hypoxia followed by reoxygenation compared to those transfected with NCX1 and NCX2 [26]. This aspect has also been the object of several in vivo studies, in which researchers employed permanent or transient occlusion of rats’ middle cerebral arteries as a model to replicate ischemic insult. The knockout of each isoform uncovered the specific involvement of NCX1 and NCX3, whose inhibition drastically extends the ischemic volume of the infarcted area, worsens the neurological deficits, and the necrotic lesion in the surviving cells of the core region and penumbra. Moreover, the upregulation of NCX3 and NCX1 mRNA in the brain regions surrounding the core insult has been interpreted as a compensatory mechanism to protect the peri-infarcted brain regions from injury [27]. In support of these data, ischemic tolerance associated with preconditioning has been correlated to the upregulation of these two gene products, NCX3 and NCX1 proteins, whose effect was proposed to be mediated by HIF1α as a transcriptional factor. The treatment consists of a short sublethal brain ischemia episode to induce adaptive transduction pathways, which in turn establish resistance. Similarly, ischemic postconditioning (IPoC) contains reperfusion complications after a prolonged harmful ischemic event, mitigating the damage and improving functional recovery. NCX3 protein and ncx3 mRNA were upregulated in those brain regions preserved by postconditioning treatment. Here, expression changes were assured by the phosphorylated form of the ubiquitously expressed serine/threonine protein kinase p-AKT, since its inhibition prevented NCX3 upregulation. NCX3 silencing, in a similar fashion, partially reverted the postconditioning-induced neuroprotection [28]. In this light, enhancing the expression and activity of NCX1 and NCX3 might represent a reasonable strategy to reduce infarct extension and improve the neurological deficits after stroke.

#### 2.3.2. Alzheimer’s Disease (AD)

Alzheimer’s disease is one of the most common neurodegenerative disorders defined by progressive memory impairment, cognitive dysfunction, and decline in language functions [29]. The neurodegenerative process is tightly linked to the accumulation of misfolded protein aggregates, resulting in deposition of β-amyloid–rich senile plaques and neurofibrillary tangles (NFTs) composed of hyperphosphorylated tau proteins. The “amyloid cascade hypothesis” posits that the abnormal accumulation and deposition of β-amyloid (Aβ) peptide in various brain areas triggers a cascade of deleterious events, including microglial activation, astrogliosis, neuroinflammation, and ionic dyshomeostasis accompanied by oxidative stress [4,30]. This pathway culminates in structural changes in neurite and cell bodies, leading to synapse loss and neuronal death. There is increasing evidence to consider the disruption of Ca^2+^ signaling because of the remodeling process caused by the massive production and deposition of Aβ peptide [4]. However, experimental models supporting the “Calcium hypothesis” have shown that the ionic imbalance occurs before the onset of symptoms, serving as an early event in the cascade leading to Aβ production and Tau hyperphosphorylation [31]. Aβ peptide disrupts Ca^2+^ signaling by enhancing the entry of external Ca^2+^ and inducing its release from intracellular stores.

Notably, the oligomers affect glutamate availability and modify NMDAR electrophysiological properties, which might culminate in NMDAR overactivation with a consequent increase in intracellular calcium concentrations [32]. Even though the exact temporal and mechanistic succession of events is yet to be fully delineated, the connection between the remodeling of ionic homeostasis and neuronal and glial responses to amyloid β1–42 (Aβ1–42)-mediated injury is undoubted.

The expression pattern of NCX1-3 is altered in the brain tissues of AD patients, as well documented by Sokolow and colleagues [33]. Compared to aged controls, the authors found a significant reduction in NCX3 protein expression in the parietal cortex and synaptosomes of brain AD patients. Also, the downregulation of this protein was accompanied by an NCX2 upregulation, probably as a compensatory mechanism. Interestingly, all three isoforms were upregulated and colocalized with Aβ oligomers in the nerve terminals [33]. This peculiar localization may be related to a protective mechanism against the Aβ-induced excitotoxic Ca^2+^ influx through hyperactivation of NMDARs, which overfunction leads to synaptic dysfunction and neuronal death, subsequent to Ca^2+^ overload. NCX1 is highly expressed in the hippocampus, cortex, and amygdala, and has also been found to enhance the NMDA-mediated Ca^2+^ influx. Its role in spatial learning and memory has been investigated employing a genetically modified mouse strain overexpressing NCX1 (ncx1.4^over^) and wild-type mice treated with CN-PYB2, a small molecule that selectively increases NCX1 activity with no effect on NCX2 and NCX3 [34].

CN-PYB2-treated mice and ncx1.4^over^ mice showed an increased performance in two tasks that require spatial learning and memory, such as the Barnes maze and context trace fear conditioning; the effect on the latter persisted for up to 4 days. On the other hand, they showed no improvement in non-spatial learning tasks, including novel object recognition tests and cued trace fear conditioning. These observations clearly lay the groundwork for investigating the therapeutic potential of CN-PYB2 through in vivo experimental models that reproduce AD (e.g., Tg2576, APP23 mice).

Since the ER has been identified as a primary site of Aβ production, the Ca^2+^ influx mediated by NCX3 working in reverse mode significantly increases the amount of Ca^2+^ ions in the ER, where the Ca^2+^ stores are depleted by the vicious circle of pathogenic Aβ production, and then ameliorates the ER stress. Piccialli et al. observed a marked enhancement of the ER Ca^2+^ content in neurons of transgenic mice (Tg2576) compared with the WT neurons, besides unchanged Ca^2+^ cytosolic levels, thus identifying the organellar Ca^2+^ dyshomeostasis as a putative biomarker of the AD pathology. However, the NCX3 reverse mode inhibition with KB-R7943 unveiled that ER Ca^2+^ release was reduced in Tg2576 neurons as well as WT hippocampal neurons. Nevertheless, this effect was significantly greater in the pathogenic condition [30].

Increased [Ca^2+^]_i_ associated with alterations in ER/mitochondria tethering establishes Ca^2+^_m_ overload as well. In this context, mitochondrial NLCX represents a new potential target in metabolic dysfunction in AD to overcome superoxide production and neuronal cell death. Besides the NCLX, the nuclear-encoded NCX3 on the outer mitochondrial membrane (OMM) participates in the Ca^2+^ handling in this organelle, suggesting that the plasma membrane isoform is not the only NCX3 that counteracts ER stress and cell death in the early stage of AD [23,24].

The studies underscore the idea that NCX3, whether plasmalemmal or mitochondrial, may exert neuroprotective effects in the context of Alzheimer’s disease (AD). Unfortunately, in the absence of selective NCX3 enhancers, these data are limited to analysis of expression changes or colocalization studies. In this light, developing small molecules able to enhance NCX3 activity selectively might be a compelling strategy to demonstrate in vivo functional rescue and validate the role of NCX3 as a therapeutic target in AD.

#### 2.3.3. Multiple Sclerosis

Multiple sclerosis (MS) is a demyelinating condition characterized by persistent inflammation, degeneration of oligodendroglia, and neuronal loss within the brain or spinal cord. A key pathological feature is episodes of neurological deficits alternating with recovery periods; the process is related to lesion genesis comprising various localized areas of myelin depletion, which are associated with differing levels of inflammation, gliosis, phagocytic activity, and damage to axons [35]. Although etiopathogenesis is still under examination, it is widely assumed that an immune response against self-myelin antigens is the main foundation; thus, the pharmacological therapies are primarily immunomodulatory. During MS progression, the proinflammatory response of microglia and the presence of infiltrating monocyte-derived macrophages significantly increase, owing to their role in promoting the oligodendrocyte precursor cells (OPCs) recruitment and differentiation in mature myelin-generating oligodendrocytes. In this respect, oligodendrocytes are responsible for producing myelin, and as such, they are the target of this abnormal immune attack. If the myelin sheath around the axon is not irreversibly damaged, it could be restored through a spontaneous regenerative process called remyelination, which would restore axonal conduction and trophic support. The process necessitates OPCs that migrate into the lesion, proliferate, and differentiate into myelinating oligodendrocytes. Remyelination failure determines the progression and severity of this neurodegenerative disorder; therefore, supporting this process represents a challenge for clinical research. The complex network of [Na^+^]_i_ and [Ca^2+^]_i_ signaling plays a pivotal role in determining whether demyelinated axons undergo repair or irreversible damage in multiple sclerosis. Research indicates that NCX1 influences monocyte-derived macrophages’ reactivity under inflammation; moreover, impaired exchangers’ reverse mode of operation contributes to pathological calcium influx, exacerbating axonal degeneration within MS lesions. However, this isoform was detected in immature OPCs, while the full characterization in mature OPCs is still lacking.

On the other hand, functional studies found a remarkably increased activity and expression of NCX3 in mature OPCs. For instance, Boscia and colleagues have shown the relevance of NCX3 in MO3.13 cells, a clonal cell line of human oligodendrocytes. Here, ncx3 overexpression is correlated with myelin markers, suggesting that increased activity may stimulate OPC differentiation and myelin synthesis in oligodendroglia [36]. In line with this study, the pharmacological inhibition of NCX3 activity in the same cell line, with a NCX3 blocker followed by drug washout, caused a rebound upregulation in NCX3 protein expression with a parallel stimulation of reverse mode activity [37].

#### 2.3.4. Parkinson’s Disease (PD)

Parkinson’s disease is a multifactorial neurodegenerative disorder in which a slow and progressive death of dopaminergic neurons occurs in the substantia nigra pars compacta and other brainstem regions. Basal ganglia neuron damage leads to typical motor symptoms: bradykinesia, tremors, postural rigidity, and instability. The physiopathology is linked to the presence of Lewy bodies, primarily composed of aggregates of the α-synuclein [38]. Accumulation of α-synuclein in human dopaminergic neurons results in different pathogenic pathways, including mitochondrial impairment via reduced activity of respiratory complex I, producing reactive oxygen species and Ca^2+^ dyshomeostasis in the organelle. The impairment of Ca^2+^ homeostasis in cytosol and mitochondrion accompanies oxidative stress, mitochondrial and proteosome dysfunction, thus it has been thoroughly documented in several models reproducing the PD’s pathophysiology. Indeed, in vitro models on human neuroblastoma SH-SY5Y cells exposed to α-synuclein and rotenone reported the dysregulation of Ca^2+^ homeostasis mediated by VGCC channels. The pharmacological administration of CGP37157, a mNCX and VGCC blocker, significantly prevented this alteration, as reported by Bastioli et al. in 2019 [39]. Contextually, Scorziello et al. pinpointed a nuclear-encoded NCX3 isoform on the outer mitochondrial membrane (OMM), which has a pivotal role in controlling Ca^2+^_m_ in the mitochondria, either in normoxic or hypoxic conditions [23,24]. Besides NCX3, also plasmalemmal NCX2 contributes to ionic balance in mitochondria, preventing neurodegeneration provoked by Ca^2+^-overload, as evidenced by a study on human dopaminergic neurons [40]. Concerning in vivo evidence, decreasing NCX3 expression and activity has been found in the midbrain neurons of A53T mice due to abnormal α-synuclein deposition, leading to ionic imbalance and mitochondrial dysfunction [41]. The latter, tightly related to disruption of calcium regulation in the organelle and cytosol, may contribute to the dysfunction of dopaminergic neurons observed in A53T transgenic mice. This event in the early stage of pathogenesis may induce proinflammatory factors’ release, leading to glial cell activation in the striatum and progressive neurodegeneration in dopaminergic neurons in the late stage [42]. Impaired NCX3 expression was accompanied by a parallel overexpression of NCX1 protein attributed to glial proliferation and microglia activation, suggesting this isoform as a useful target to overcome neuroinflammation in patients.

#### 2.3.5. Amyotrophic Lateral Sclerosis (ALS)

The neuropathological hallmark of amyotrophic lateral sclerosis (ALS) is a progressive and selective death of motor neurons in the brain and spinal cord, leading to progressive paralysis of voluntary muscles. Although the exact cause of ALS remains elusive, extensive research has shed light on the complex interplay of various pathological mechanisms that drive this relentless disease. Oxidative stress, stemming from an imbalance in excessive reactive oxygen species (ROS) production and inadequate antioxidant defenses, is a critical element for motor neuron degeneration in ALS. Mitochondria, as the primary source of cellular energy, are particularly vulnerable to oxidative damage, aggravating neurodegeneration [43]. Genetic mutations, such as C9orf72 and sod1, and environmental factors like exposure to toxins can exacerbate oxidative stress, further accelerating the disease progression [44]. SOD1 stands for Superoxide Dismutase 1, an essential antioxidant enzyme primarily found in the cytoplasm and mitochondrial intermembrane space of cells. Mutations of the SOD1 gene are tightly linked to the familial form of ALS, in which the motor neuron toxicity is mainly caused by deposition of misfolded proteins and their accumulation, rather than enzyme activity impairment. However, an interesting study using transgenic mice with mutant SOD1G93A underlined that a rapid exposure to wild-type SOD1 might counteract the ER stress elicited by the beta methylamino-L-alanine (L-BMAA) administration [45]. Excitotoxicity, characterized by excessive stimulation of glutamate receptors, leads to an abnormal calcium influx into motor neurons. In this scenario, calcium overload triggers a cascade of events, including mitochondrial dysfunction, toxic accumulation of misfolded proteins, and ROS generation, which create a self-perpetuating cycle of neuronal degeneration. Indeed, ALS patients show higher calcium levels both in muscle and in spinal cord muscle [46]. The involvement of NCX in ALS has been investigated by Anzilotti et al. in SOD1G93A mice, finding a close connection between protective preconditioning and the third isoform of the exchanger. Preconditioning is a neuroprotective strategy induced by a subthreshold dose of the neurotoxin L-BMAA, responsible for ALS induction [47]. The observed delay in motoneuron degeneration and the prolonged survival have been linked to the neuroprotection elicited by the prevention of NCX3′s reduced expression in the CNS and muscles. Moreover, among all the NCX proteins, the third isoform is predominantly expressed in skeletal muscles and at lower levels in the CNS, supporting the possible involvement in ALS pathogenesis and, above all, its druggability [48]. Furthermore, NCX1 has been identified as a key mediator of the non-enzymatic neuroprotective effect of SOD1 in an in vitro model of ALS. Under normal conditions, NCX1 co-localizes with endogenous SOD1 at the plasma membrane of motor neurons. Functionally, both SOD1 and its apo form trigger NCX1 activation in reverse mode, promoting Ca^2+^ influx through increased intracellular Na^+^. This facilitates ER Ca^2+^ refilling, prevents ER stress, and induces Akt activation and nuclear translocation in motor neurons. Accordingly, pharmacological stimulation of NCX1 with CN-PYB2 mitigates L-BMAA-induced neurotoxicity, supporting its potential as a novel therapeutic target for ALS [49].

#### 2.3.6. NCX in Neuroplastic Diseases of CNS: Glioblastoma

Gliomas are malignant brain tumors originating from glial cells and are the most common type of primary central nervous system tumors in adults. Glioblastoma, also known as glioblastoma multiforme (GBM), is a highly aggressive and fast-growing type of brain tumor that originates from glial cells, specifically astrocytes, which support nerve cells in the brain. It represents 50% of all gliomas, and it is roughly the most aggressive form of a malignant brain tumor in adults, characterized by rapid progression, resistance to treatment, and a poor prognosis, with limited survival rates even after aggressive therapy such as surgery, radiation, and chemotherapy due to its invasiveness [50]. Tumorigenesis is multifactorial; numerous genetic mutations have been found in GMB patients, along with metabolic and ionic alterations related to cancer progression. Also, mutations impacting ion channels and pumps, discovered in 90% of GBM cases, play a key role in driving the rapid proliferation, migration, and invasiveness of tumor cells, deserving the description of “oncochannels” [51,52]. While various alterations in Ca^2+^ transporters, pumps, and channels have been investigated in cancer cell models, the role of NCX in the ionic changes observed in tumor cells remains largely underexplored. The currently available data suggests that channels and exchangers might be involved in cell invasion through two main mechanisms: building a hyperosmotic environment with increased cell volume at the leading edge, and cell shrinkage. The posterior edge of the cell experiences shrinkage due to the efflux of salts, which decreases cytosolic water content. Current theories indicate that NKCC (Na^+^-K^+^-Cl^−^ cotransporter) and Na^+^-K^+^ ATPase contribute to the accumulation of Cl^−^ and K^+^ ions within the cells. This accumulation generates the necessary electrochemical driving force to initiate these osmotic changes [53,54].

A recent study in human GBM cell lines (U251, U87, GSCs) has highlighted the high NCX expression in lamellipodia of migrating cells, indicating its role in this cellular structure. The accumulation of ions such as Na^+^ and Ca^2+^ through NCX generates the electrochemical gradients required for osmotic changes that facilitate cell shape alterations at the leading edge, promoting lamellipodia extension. Inhibition of NCX leads to impaired lamellipodia formation and maintenance, which consequently disrupts the migration of GBM cells. This suggests that NCX is not only present but also vital for the dynamic processes involved in cell movement [54].

The antiproliferative activity of NCX has scarcely been reported, maybe because the specific NCX expression pattern is controversial in glioblastoma cell lines. In this regard, an interesting point of view was provided by the antiproliferative activity of SKF 96365 employed in a concentration range like that applied for TRPC blockade. SKF 96365 inhibited the growth of glioblastoma cells by directly enhancing the reverse mode of NCX, owing to the increase in intracellular calcium levels. Notably, the study has revealed that NCX1 isoform expression was significantly elevated in glioblastoma cells compared to normal astrocytes. The NCX1-knockout in glioblastoma cells reduced the effectiveness of SKF 96365, indicating that NCX represents a promising target for inhibiting glioblastoma cell proliferation [55].

Following this path, Hu and coll. in 2019 [56] have described that inhibiting the NCX forward mode using selective blockers like bepridil, CB-DMB, or KB-R7943 can increase intracellular calcium ([Ca^2+^]_i_) levels and induce cell death in glioblastoma (GBM) cells. Specifically, bepridil and CB-DMB were found to cause calcium-dependent cell cycle arrest and apoptosis in GBM cells, which were attenuated by the calcium chelator BAPTA-AM [56]. Interestingly, bepridil did not disclose a cytotoxic effect on human astrocytes, expressing higher levels of functional NCX than GBM cells. The low expression of NCX in GBM cells makes them particularly sensitive to disturbances in intracellular calcium homeostasis. These findings suggest that interventions targeting the forward mode of NCX could have therapeutic potential by inducing calcium-mediated injury, specifically in GBM cells.

Current knowledge does not fully clarify the role of NCX in the progression of glioblastoma; furthermore, inhibiting the forward mode or enhancing the reverse mode of NCX may lead to an increase in the intracellular calcium levels, which could initiate cell death mechanisms in tumor cells. Thus, it is worth exploring the modulation of the abnormal activity of NCX isoforms, which are selectively expressed in cancer cells, to disrupt intracellular Ca^2+^ balance and potentially hinder the progression of glioblastoma (GBM).

**Table 1:** Summary of key findings on NCX isoforms (NCX1, NCX2, NCX3) in various neurological diseases, including cerebral ischemia, Alzheimer’s disease, multiple sclerosis, Parkinson’s disease, ALS, and glioblastoma. Study type and references are indicated for each observation.

**Cerebral Ischemia**	**Study Type**	**Ref**
NCX3 is minimally affected by ATP depletion and helps maintain Ca^2+^ homeostasis in ischemia-like conditions. BHK cells transfected with NCX3 showed greater resistance to hypoxia/reoxygenation than those with NCX1 or NCX2.	In vitro	[26]
NCX1/NCX3 knockout worsens infarct size and neurological outcome. Upregulation of NCX3 and NCX1 mRNA in peri-infarcted regions interpreted as a protective mechanism.	In vivo (rat model)	[27]
Ischemic preconditioning upregulates NCX3 and NCX1 via HIF1α, enhancing ischemic tolerance.	In vivo (preconditioning)	[27]
Postconditioning (IPoC) induces upregulation of NCX3 protein and mRNA in protected brain regions via p-AKT; NCX3 silencing reduces this effect	In vivo (postconditioning in rats)	[28]
**Alzheimer’s Disease**	**Study Type**	**Ref**
Calcium hypothesis suggests that ionic imbalance precedes symptoms and promotes Aβ and Tau pathology.	Experimental models	[31]
Aβ oligomers enhance NMDAR activity, increasing intracellular Ca^2+^ levels and excitotoxicity.	Experimental models	[32]
NCX3 is significantly downregulated in AD parietal cortex and synaptosomes; NCX2 is upregulated. All three NCX isoforms colocalize with Aβ oligomers at nerve terminals, suggesting a protective mechanism.	Human post-mortem tissue study	[33]
NCX3 may help refill ER Ca^2+^ stores, thus alleviating Aβ-induced ER stress.	Hypothesis/literature evidence	[4]
**Multiple Sclerosis**	**Study Type**	**Ref**
NCX3 expression and activity are significantly increased in mature OPCs (e.g., MO3.13 human oligodendrocyte cells). NCX3 overexpression in MO3.13 cells correlates with myelin markers and may promote OPC differentiation and myelin synthesis.	In vitro (MO3.13 cells)	[36]
NCX3 pharmacological inhibition followed by washout causes NCX3 upregulation and enhanced reverse mode activity.	In vitro (MO3.13 cells)	[37]
**Parkinson’s Disease**	**Study Type**	**Ref**
In SH-SY5Y cells treated with α-synuclein and rotenone, Ca^2+^ dysregulation occurs via VGCCs; CGP37157 (mNCX/VGCC blocker) prevents this.	In vitro (SH-SY5Y cells)	[39]
NCX3 is located on the outer mitochondrial membrane and regulates mitochondrial Ca^2+^ under normoxic/hypoxic conditions.	Literature review/Experimental	[23,24]
NCX2 at the plasma membrane helps maintain mitochondrial ionic balance and prevent neurodegeneration.	In vitro (human dopaminergic neurons)	[40]
NCX3 downregulation in midbrain of A53T mice causes mitochondrial dysfunction and calcium imbalance.	In vivo (A53T transgenic mice)	[41]
Disrupted calcium homeostasis induces neuroinflammation and progressive neurodegeneration. NCX1 upregulation in glia may be a compensatory response to neuroinflammation; potential therapeutic target.	In vivo (A53T mice)	[42]
**Amyotrophic Lateral Sclerosis (ALS)**	**Study Type**	**Ref**
In SOD1G93A mice, wild-type SOD1 exposure counteracts L-BMAA-induced ER stress.	In vivo (transgenic mice)	[45]
NCX3 is involved in neuroprotection in ALS; its expression is preserved via L-BMAA preconditioning in SOD1G93A mice.	In vivo (SOD1G93A mice)	[47]
NCX1 mediates SOD1-induced neuroprotection via reverse mode Ca^2+^ influx, ER refilling, Akt activation. NCX1 stimulation (e.g., CN-PYB2) reduces L-BMAA toxicity, suggesting therapeutic relevance.	In vitro (ALS model)	[49]
**Glioblastoma**	**Study Type**	**Ref**
NCX is highly expressed in lamellipodia of GBM cells; NCX inhibition disrupts lamellipodia and cell migration, highlighting its role in motility.	In vitro (U251, U87, GSCs)	[54]
SKF 96365 induces reverse NCX mode, increasing intracellular Ca^2+^ and reducing GBM cell proliferation. NCX1 is upregulated in GBM cells vs. astrocytes; NCX1 knockout reduces SKF 96365 efficacy.	In vitro	[55]
Bepridil, CB-DMB, KB-R7943 (NCX forward mode blockers) raise [Ca^2+^]_i_ and induce GBM cell death. These effects are calcium-dependent and selective for GBM cells (not astrocytes), suggesting therapeutic window.	In vitro	[56]

## 3. Targeting NCXs with Heterocyclic Compounds

In recent decades, pharmacological modulation of the Na^+^/Ca^2+^ exchanger (NCX) using organic molecules has emerged as a promising avenue of research, primarily due to its essential role in regulating calcium homeostasis across diverse cell types. Recognizing the importance of NCX in cardiac contractility and the nervous system, several research groups have developed organic compounds designed to selectively inhibit or activate specific NCX isoforms. These pharmacological agents not only enhance our understanding of NCX’s functional dynamics, but they also have therapeutic potential for treating health issues, such as heart disease, neurological disorders, and cancer.

Table 2, presented at the end of Section 3, offers a concise and accessible overview of the reported studies. It summarizes the key pharmacological properties of selected compounds acting on Na⁺/Ca²⁺ exchangers NCX1, NCX2, and NCX3, including isoform selectivity, representative IC₅₀/EC₅₀ values, and reversibility.

### 3.1. Inhibitors

A gamut of NCX inhibitors has been developed to improve drug selectivity and potency toward NCX isoforms. These inhibitors include various chemical classes, such as benzyloxyphenyl analogs, quinazolinones, thiazolidines, phenoxy pyridines, acetamides, benzofurans, and imidazolines. Notably, some of these compounds demonstrate greater selectivity for either the forward or reverse mode of NCX operation, a phenomenon influenced by the differing intracellular sodium concentrations ([Na^+^]_i_) used in measuring NCX activity.

#### 3.1.1. Amiloride Derivatives

**Figure 3:**
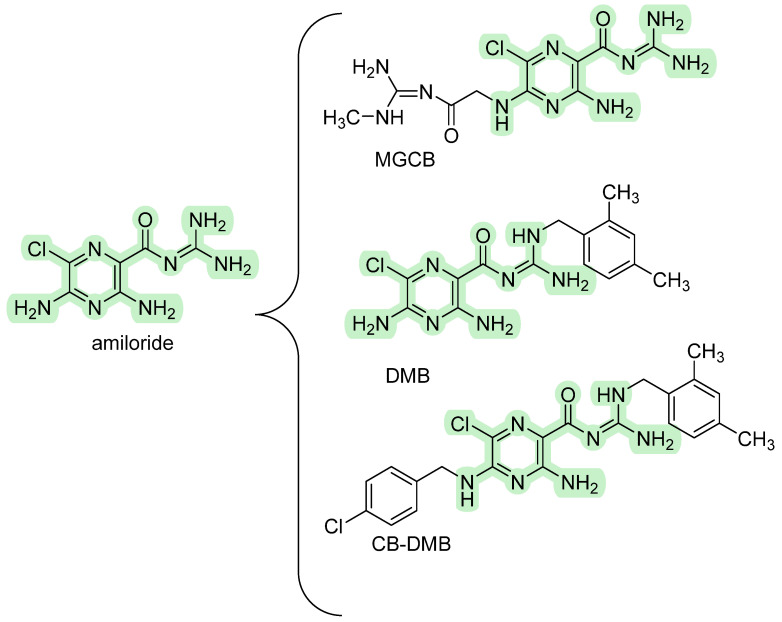
Structures of the most NCX-inhibiting amiloride derivatives.

Several derivatives structurally related to amiloride have been extensively investigated for NCX’s inhibitory activity due to their potential use to assess the implication of this target in complex disorders [49,57,58,59]. The pharmacological profile involves various sodium transport systems, including NCX, epithelial sodium channels (ENaC), and Na^+^/H^+^ exchangers (NHE), and it varies remarkably based on structural modifications. Starting from the amiloride feature, significant efforts have been devoted to developing more selective analogs (shown in Figure 3).

Among them, the first-in-class is 5-[N-methyl-N-(guanidine-carbonylmethyl)] benzamil-amiloride (MGCB), which bears substituents on the 5-amino nitrogen atom of the pyrazine ring. Notably, this compound does not exhibit inhibitory effects on the epithelial Na^+^ channel (ENaC) or NCX, although it demonstrates increased effectiveness in inhibiting Na^+^/H^+^ exchanger (NHE) within the 1–10 μM range. The second class includes compounds that do not inhibit the NHE and have substituents on the terminal guanidino nitrogen atom acting as specific inhibitors (K_i_ values of 1–10 μM) of ENaC and NCX. Among these, dimethylbenzamyl-amiloride (DMB) and 5-(N-4-chlorobenzyl)-2′,4′-dimethylbenzamil-amiloride (CB-DMB) show no inhibitory effects against NHE and ENaC in excitable cells but effectively inhibit NCX isoforms with varying degrees of potency [60]. Specifically, the IC_50_ values for inhibiting NCX1, NCX2, and NCX3 isoforms fall within the nanomolar concentration range. Additionally, this compound inhibits both the forward and reverse modes of operation for each isoform. Interestingly, the potency of CB-DMB is only slightly lower than that of YM-244769, which is currently the most potent available inhibitor for NCX1, NCX2, and NCX3. Furthermore, they share a reversible inhibition of NCX activity, and this phenomenon is competitive concerning Na^+^ ions. Thus, it has been hypothesized that these derivatives function as Na^+^ analogs and interact at a Na^+^ binding site of the NCX molecule, binding the transporter in an inactive complex.

#### 3.1.2. Benzyloxyphenyl Inhibitors

**Figure 4:**
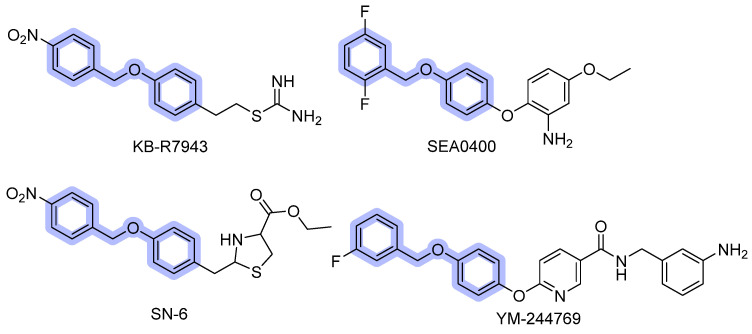
NCX’s inhibitors with benzyloxyphenyl scaffold.

KB-R7943, SEA0400, SN-6, BED, and YM-244769, here classified in the benzyloxyphenyl group (Figure 4), have been extensively studied and are currently considered to be selective NCX inhibitors (some at low doses), having a fascinating selectivity profile towards the different isoforms. KB-R7943, 2-[2-[4-(4-nitrobenzyloxy)phenyl]ethyl]isothiourea methane sulfonate, was the first synthesized NCX inhibitor. Its pharmacological profile has been widely investigated for the effects on NCX activity in both cardiac and neuronal tissues. As evidenced by Iwamoto and colleagues in many studies [61,62], KB-R7943 does not inhibit NCX currents when applied internally through a pipette solution, suggesting an interaction with an external binding site on the exchanger. Indeed, the action on the α_2_ repeat, between the TM7 and TM8, leads to a rapid inhibition of outward NCX currents exhibiting selectivity for the reverse mode of operation [63]. Mutations at Val820, Gln826, and Gly833 residues significantly affect KB-R7943′s inhibition, as a consequence of these residues’ role in forming the drug-binding site [64]. Even though KB-R7943 blocks the reverse mode activity of NCX at low doses (≥0.8 µmol/L) in concentrations up to 10 µM, it also inhibits other ion channels, such as Na^+^/H^+^ exchanger, Na^+^-K^+^ ATPase, NMDA receptors, and N-type, P/Q-type, and L-type Ca^2+^ channels [65,66,67]. In addition, KB-R7943 is also a potent blocker of TRPC channels (IC_50_ 0.46, 0.71, and 1.38 μM at TRPC3, TRPC6, and TRPC5, respectively).

Recently, Andreeva-Gateva et al. have conducted a pilot pharmacokinetic (PK) study that supported the promising neuropharmacological activity of orally administered KB-R7943 [68]. The compound showed rapid absorption (T_max_ ~ 1 h), measurable heart and brain penetration, prolonged tissue exposure up to 24 h, and urinary excretion partly unmetabolized.

SEA0400, 2-[4-[(2,5-difluorophenyl)methoxy]phenoxy]-5-ethoxybenzenamine, was first reported in 2001 by Matsuda [69]. and compared with KB-R7943. This inhibitor is highly specific for NCX, preventing the Na^+^-dependent Ca^2+^ uptake in cultured neurons, astrocytes, and microglia with IC_50_ of 33 nM, 5.0 nM, and 8.3 nM, respectively. Moreover, it is inadequate to inhibit other receptors, channels, and transporters [70,71].

In 2002, screening new benzyloxyphenyl derivatives led to the discovery of SN-6, 2-[4-(4-nitrobenzyloxy)benzyl]thiazolidine-4-carboxylic acid ethyl ester, which showed similar inhibitory potency for NCX1 to the previous one but was more specific for NCX1 [72,73]. These inhibitors have shown different isoform selectivity: KB-R7943 is threefold more effective on NCX3 than on NCX1 or NCX2, whereas SEA0400 predominantly blocks NCX1, only mildly blocks NCX2, and exerts almost no influence upon NCX3. SN-6 is up to fivefold more inhibitory to NCX1 (IC_50_ 2.9 μM) than NCX2 (IC_50_ 16 μM) and NCX3 (IC_50_ 8.6 μM). Further site-directed mutagenesis was employed to shed light on the critical residues involved in drug sensitivity. It turned out that Gly833 and Ans839 in NCX1 are common molecular determinants required for inhibition by all benzyloxyphenyl derivatives. On the other hand, Phe213 and Val227/Tyr228 are specific determinants involved in the molecular inhibition operated by SEA0440 and SN-6. The Gly833 residue in the NCX3 structure is also relevant to the effect of the compound N-(3-aminobenzyl)-6-[4-[(3-fluorobenzyl)oxy]phenoxy] nicotinamide named YM-244769, which is 80 times more potent than KB-R7943 in inhibiting the reverse mode of NCX3 (IC_50_ 18 nM). Of note, a preferential activity for NCX3 was observed, compared to other isoforms (IC_50_ values 68 nM and 96 nM for CCL39 cells transfected with NCX1 and NCX2) [74,75,76].

**Figure 5:**
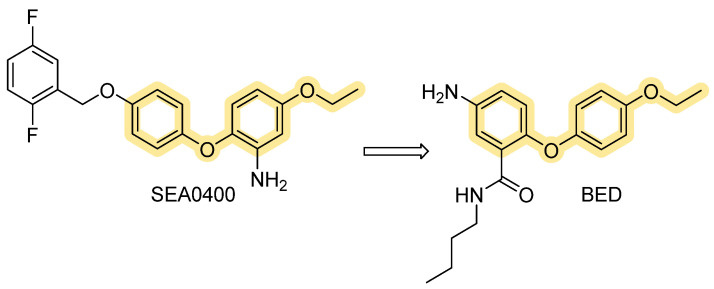
Chemical structure of SEA0400 and BED.

The chemical modification of the biphenyl ether moiety of SEA0400 resulted in BED’s design (Figure 5), the 5-amino-N-butyl-2-(4-ethoxyphenoxy)-benzamide hydrochloride, a more recent benzyloxyphenyl derivative, originated from the insertion of an amide functional group. These changes are strongly reflected in biological activity; this molecule has shown, in fact, a significantly higher potency than other NCX inhibitors so far described (IC_50_ = 1 nM). Moreover, in contrast to those previously described, this compound can inhibit NCX3 either in reverse or forward mode [77,78].

Research in this field is still in progress, in fact, a recent cryo-EM study on human NCX1 by Dong et al. [79]. reports that SEA0400 binds an allosteric site, limiting conformational rearrangements associated with ion exchange and effectively reducing calcium uptake activity. The binding pocket is distinct from ion passage, suggesting that SEA0400 inhibits NCX1.3 through an allosteric mechanism rather than direct competition at ion binding sites. From a functional point of view, most benzyloxyphenyl compounds mainly block the Ca^2+^ influx mode of NCX. Due to their affinity profile, they should be properly used depending on the target organs, which express specific NCX isoforms.

#### 3.1.3. Quinazolinone Derivatives

**Figure 6:**
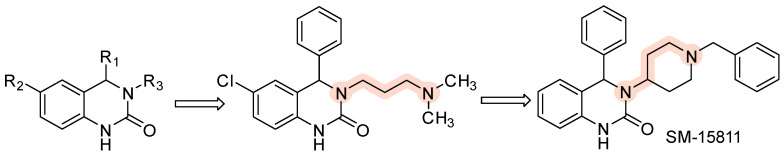
Discovery of SM-15811, a new 3,4-dihydro-2(1H)-quinazolinone derivative.

SM-15811 is the only noteworthy molecule in this chemical class. The 3,4-dihydro-2(1H)-quinazolinone derivative has demonstrated a concentration-dependent alleviation of the increase in Na^+^-free-induced Fura 2 fluorescence ratio and exhibited inhibitory activity against the Na^+^/Ca^2+^ exchanger with an IC_30_ value of 0.46 µM [80]. The SAR study that led to this compound (simplified in Figure 6), which was raised from a random screening of a chemical library, indicated that the 3,4-dihydro-2(1H)-quinazolinone scaffold with a tertiary aminoalkyl side chain at the 3-position was critical for high activity. Moreover, the addition of a 4-substituted piperidine moiety, particularly the 4-benzylpiperidin-1-yl group, significantly enhanced inhibitory potency against the Na^+^/Ca^2+^ exchanger [81]. Interestingly, SM-15811 shows excellent selectivity for the Na^+^/Ca^2+^ exchanger over other ion channels, such as Na^+^ and K^+^ channels. This selectivity reduces the likelihood of off-target, suggesting employment against myocardial ischemia–reperfusion injury, where it might help to preserve heart tissue by mitigating calcium-induced damage.

#### 3.1.4. Flavan and Furan Derivatives

**Figure 7:**
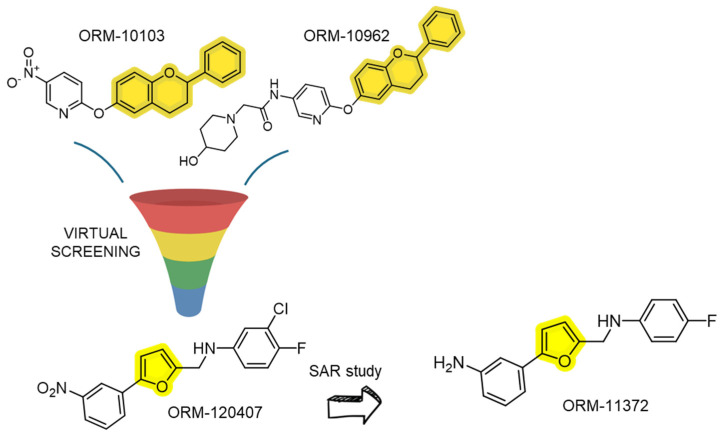
Discovery of ORM-11372, an NCX1.1 inhibitor, via virtual screening and medicinal chemistry optimization.

These chemical classes emerged from the virtual screening using two flavan-based pharmacophore models, already known as ORM-10103 and ORM-10962 [82]. Following the results of the virtual screening, the authors proceeded with the chemistry optimization of the lead compound until obtaining the furanic derivative ORM-11372 (as depicted in Figure 7). The hit, ORM-120407, demonstrated an NCX1 inhibitory activity on the order of 200 nM. However, it lacked sufficient selectivity against other ion channels, such as hERG (human ether-à-go-go-related gene, IC_50_ 2.1 μM) and L-type calcium channels (IC_50_ 3.1 μM), necessitating further structural refinement. The lead compound has been examined for structure–activity relationship leading to ORM-11372, the first in the class of the third generation of NCX1.1 inhibitors with a unique scaffold and improved profile. The interesting inhibitory effect (IC_50_ of 6 nM) was also accompanied by a promising selectivity towards hERG (IC_50_ 10.6 μM) and L-type Ca^2+^ channels (IC_50_ 6.1μM), even if the NCX subtype selectivity is still unknown. The scaffold of ORM-11372 is defined by a bridged aniline structure that allows for hydrogen bonding between the A- and B-ring systems, a feature absent in first- and second-generation NCX inhibitors. This unique structural element likely contributes to the increased potency observed in screening assays compared to known NCX inhibitors, hinting at a specific binding interaction between ORM-11372 and the NCX protein. However, this hypothesis remains tentative due to the lack of direct experimental validation, such as protein–ligand crystal structures. Therefore, concrete evidence of the binding pocket for ORM-11372 and other NCX inhibitors is still lacking.

#### 3.1.5. 1,4-Benzothiazepines

The regulation of calcium outside the mitochondria occurs through two sequential processes involving the Na^+^-sensitive NCLX. This exchanger can transport either Li^+^ or Na^+^ in exchange for Ca^2+^, effectively facilitating the transfer of Ca^2+^ from the mitochondrial matrix to the intermembrane space. Subsequently, mNCX3 contributes to this process by promoting the efflux of Ca^2+^ from the intermembrane space into the cytosol, ensuring proper calcium homeostasis within the cell. Mitochondria are unable to retain calcium, as prolonged and sustained levels of mitochondrial calcium concentration ([Ca^2+^]_m_) can induce the opening of the mitochondrial permeability transition pore, leading to the initiation of apoptotic pathways. Consequently, the mitochondrial Na^+^/Ca^2+^ exchanger (mNCX) plays a critical role in controlling [Ca^2+^]_m_ and enabling calcium communication among the mitochondria, the cytosol, and the endoplasmic reticulum (ER) [83,84].

**Figure 8:**
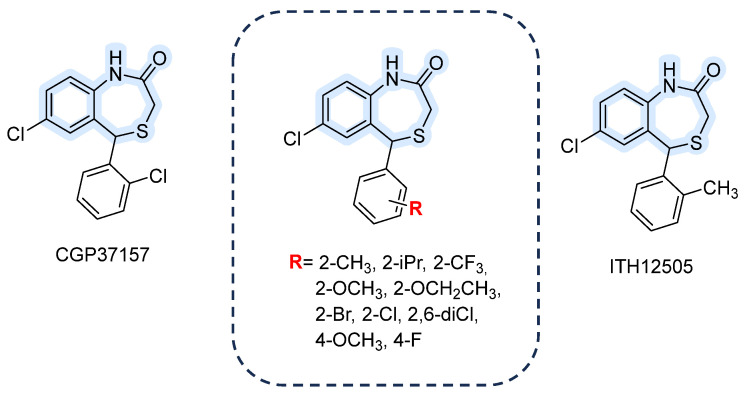
Benzothiazepine CGP37157 derivatives.

The 7-chloro-5-(2-chlorophenyl)-3,5-dihydro-4,1-benzothiazepin-2-(1H)-one, known as CGP37157, is widely used as a pharmacological tool to study mNCX implications in many disorders, such as diabetes or neurodegenerative disease [85,86]. This molecule demonstrated a blocking activity 20 times greater than previously studied ligands (IC_50_ 0.8 μM in Na^+^-induced Ca^2+^-release from guinea-pig heart mitochondria). For instance, it still represents the reference ligand for investigating the mitochondrial sodium/calcium exchanger (mNCX) even though many derivatives have been developed (structures shown in Figure 8) [87].

ITH12505 is an isosteric analog, developed by Gonzalez-Lafuente et al., in which the chlorine at the C2′ position of the phenyl ring is replaced with a methyl group [88]. In 2012 they compared ITH12505 with CGP37157, finding that both compounds provided neuroprotection in hippocampal slices subjected to glutamate excitotoxicity in a concentration-dependent manner, with maximal protection at 30 µM. Notably, ITH12505 protected SH-SY5Y cells under oligomycin A/rotenone stress, where CGP37157 showed no effect. In hippocampal slices undergoing oxygen/glucose deprivation followed by reoxygenation, ITH12505 was protective at concentrations of 3–30 μM, whereas CGP37157 was only effective at 30 μM. An in vitro parallel artificial membrane permeability assay for the blood–brain barrier (PAMPA-BBB) confirmed that both compounds can cross the BBB, allowing them to reach their central nervous system targets [88]. Also, unlike CGP37157, which caused a marked reduction in cell viability at 30 μM, ITH12505 demonstrated a broader therapeutic window, showing no toxicity in SH-SY5Y cells even at micromolar concentrations. Hence, with the well-established VGCC (voltage-gated Ca^2+^ channels) blocking action of CGP37157 and the enhanced selectivity profile achieved for ITH12505 through additional scaffold pharmacomodulation, it becomes evident that there is a compelling demand to develop alternative analogs that exhibit greater potency and selectivity for mNCX.

### 3.2. Activators

Compared to the variety of organic molecules blocking the Na^+^/Ca^2+^ exchanger, the research on the activators’ field is still significantly lacking. It has been reported that several agonists of G-protein-coupled receptors, such as α- and β- receptors, histaminergic, serotoninergic 5HT2A, and angiotensin-II receptors, may stimulate the sodium/calcium exchanger by the pathway governed by PKA and protein kinase C. Undoubtedly, using this molecule as a tool to investigate or address pathophysiological disorders involving ionic dyshomeostasis is limited, especially if NCX is directly implicated. Therefore, there is a growing interest in discovering selective activators targeting the various isoforms mentioned above.

#### The 1,4-Benzodiazepine-2-One Analogs

**Figure 9:**
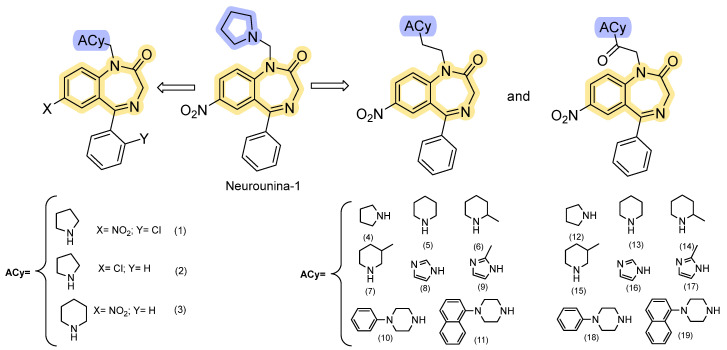
Neurounina-1 analogs [91].

To develop an activator for NCX isoforms, Molinaro and colleagues modified the structure of SM-15811, one of the most potent inhibitors. In their approach, they replaced the 3,4-dihydro-2(1H)-quinazolinone scaffold with a seven-member heterocyclic ring, while maintaining the nitrogen atoms and the ketone group [89]. The newly discovered compound, 7-nitro-5-phenyl-1-(pyrrolidin-1-ylmethyl)-1H-benzo[e][1,4]diazepin-2(3H)-one, known as Neurounina-1, has demonstrated highly promising pharmacological activity (chemical structure in Figure 9). Neurounina-1 effectively and reversibly enhances the activity of NCX1 and NCX2 in a concentration-dependent manner, showing remarkable potency in the low nanomolar range (0.03–2.7 nM). The compound displayed more efficiency in NCX1′s forward mode (Ca^2+^ efflux) than NCX2, remaining substantially ineffective on the NCX3 isoform even at concentrations up to 10 µM.

The drug response lies in the α_1_ and α_2_ segments, where Val118, Asn125, and Leu808 are involved in NCX1 responsiveness [89]. Even though Neurounina-1 contains a privileged scaffold, it does not activate GABA_A_ receptors at concentrations adequate for the antiporter, as the authors observed an absence of influence on GABA-induced chloride currents [78]. The potential neuroprotective effect was investigated in an experimental model of cerebral ischemia, where Neurounina-1 significantly reduces infarct volume in adult mice subjected to transient middle cerebral artery occlusion, and in newborn mice exposed to hypoxia. Remarkably, the neuroprotective effects of a single dose of Neurounina-1 can last for an extended period, remaining observable up to one month post administration. A sensitive and selective LC-MS/MS method (LLOQ 1 ng/mL) enabled high-throughput quantification of Neurounina-1 in beagle dog plasma. Following single intravenous doses (0.1–1 mg/kg), linear pharmacokinetics were observed. Despite high lipophilicity, Neurounina-1 showed low half-life (≈2–2.5 h) with CL of 46–69 L/h and V_d_ of 130–211 L [90]. The attractive pharmacological profile of Neurounina-1, in concern with its striking pharmacokinetic and pharmacodynamic properties, has inspired the design of nineteen analogs through traditional pharmacomodulation, building up our comprehension of the protein’s structure dynamics. Magli et al. [91] developed a new small library of NCX modulators (Figure 9), in which the 1,4-benzodiazepine-2-one nucleus is linked to five- or six-member cyclic amines via a methylene, ethylene, or acetyl spacer. Conformational and steric properties were analyzed to determine the molecular volume required for selectively activating/inhibiting NCX1, NCX3, or both [91]. Using the prokaryotic Na^+^/Ca^2+^ exchanger (NCX_Mj) as the exchanger’s structural model, the authors have supposed that NCX activators should stabilize the OF calcium-loaded open conformation (reverse mode) and the OF sodium-loaded open conformation (forward mode).

Specifically, the activators may stabilize a specific relative orientation between the core domain and the gating bundle that corresponds to the open state of the channel. Stabilization is crucial for maintaining the transporter in a conformation that allows for ion passage. Otherwise, the interaction with TM2 and TM7 instead of TM1-6 can weaken the connection between the core domain and the gating bundle. The altered interactions lower the energetic barrier (ΔG) associated with the transition from the closed (IF) to open (OF) state, facilitating easier movement into the open conformation. From a functional point of view, the pharmacological evaluation shed some light on NCX stimulation in neuroprotection. Firstly, the research results indicate that the neuroprotective profile is more closely associated with the stimulation of NCX1 and NCX3′s reverse mode (Ca^2+^ entry) rather than an enhanced ability to promote NCX3′s forward mode (Ca^2+^ efflux). Additionally, the SAR of 1,4-benzodiazepin-2-one derivatives has provided new insights for selectively targeting disease-related NCX variants pharmacologically.

**Table 2:** This table summarizes key pharmacological properties (isoform selectivity, representative IC₅₀/EC_50_ values, reversibility) for selected chemical compounds acting on Na⁺/Ca²⁺ exchangers NCX1, NCX2 and NCX3. IC₅₀/EC_50_ values depend strongly on assay type, species, splice-variant and whether forward or reverse mode was tested. Consult the cited references for exact experimental conditions.

Cmp	Isoform Selectivity	Representative IC_50_/EC_50_	Reversibility	Ref.
Amiloride	Weak, non-selective NCX blocker	≈100 µM–1 mM (reverse mode; highly assay-dependent)	Reversible; many off-targets (NHE1, ENaC, ASICs).	[2]
CB-DMB	Pan-NCX inhibitor (blocks NCX1/2/3 bidirectionally)	Nanomolar–low µM for outward/inward components reported (assay-dependent).	Reversible; mutation mapping suggests interaction near the f-loop. Useful pan-NCX tool.	[60]
KB-R7943	NCX1 reverse-mode	1.2–2.4 µM (Na_i_^+^-dependent ^45^Ca^2+^ uptake and Na_i_^+^-dependent [Ca^2+^]_i_ increase in cardiomyocytes, smooth muscle cells, and NCX1-transfected fibroblasts) 4.3 µM (Na^+^ independent ^45^Ca^2+^ uptake in fibroblasts)	Reversible; promiscuous off-target profile (NMDA, mitochondrial complex I, other channels).	[64,65,66,67,92]
SEA0400	Strong preference for NCX1 > NCX2/3; inhibits both modes but reverse stronger	NCX1 ≈ 53 nM; NCX2 ≈ 0.98 µM (Na_i_^+^-dependent ^45^Ca^2+^ uptake into stable transfected CCL39 cells); IC_50_ of 33 nM, 5.0 nM and 8.3 nM (Na^+^-dependent Ca^2+^ uptake in cultured neurons, astrocytes, and microglia)	Reversible; more selective and potent than KB-R7943 but assay-dependent.	[69,70,71]
SM-15811	Potent NCX1 inhibition; structure modified to obtain Neurounina-1 activator	IC_30_ = 0.46 µM (Na^+^-free-induced Fura 2 microfluorimetry)	Reversible	[80]
SN-6	Selective for NCX1 reverse mode; negligible on NCX2/3	NCX1 ≈ 2.3–2.9 µM; NCX2 ≈ 16 µM; NCX3 ≈ 8.6 µM. (Na_i_^+^-dependent ^45^Ca^2+^ uptake into stable transfected CCL39 cells)	Reversible, improved selectivity over SEA0400	[72,93]
YM-244769	Preferential potency for NCX3 (NCX3 > NCX1 > NCX2)	NCX3 ≈ 18 nM; NCX1 ≈ 68 nM; NCX2 ≈ 96 nM (Na_i_^+^-dependent ^45^ Ca^2+^ uptake into stable transfected CCL39 cells)	Reversible; used to probe NCX3 contribution in neuronal models.	[73]
BED	Potent NCX3 inhibitor (used to interrogate NCX3 roles)	NCX3: ~ 1.9 nM; NCX2 ~ 3.5 nM (Na_i_^+^-dependent ^45^Ca^2+^ uptake and Na_0_^+^-dependent ^45^Ca^2+^ efflux in stably transfected BHK cells).	Reversible; reported to worsen anoxic injury in cortical neurons when NCX3 inhibited.	[74]
ORM-10103/ORM-10962	Designed as selective NCX inhibitors	Sub-µM to nM range in whole-cell cardiac NCX assays.	Reversible; improved selectivity vs. earlier scaffolds; abolishes triggered arrhythmias in models.	[94,95]
ORM-11372	Very potent on human NCX1.1	EC_50_ (human NCX1.1): reverse ≈ 5 nM; forward ≈ 6 nM (on human-induced pluripotent stem cell (iPSc)-derived cardiomyocytes).	Reversible; optimized drug-like profile, profiled for cardiac safety.	[82]
CGP-37157/ITH12505	Inhibit mitochondrial Na^+^/Ca^2+^ exchange; also affect plasma membrane NCX at higher conc.	Active concentrations low-µM depending on system (mitochondrial vs. plasma assays).	Reversible; not fully selective—affect VGCCs and other Ca^2+^ handling pathways at similar concentrations.	[87,88,96]
Neurounina-1 and derivatives	Functional activation of NCX1 and NCX2	Allosteric activation of NCX1 and NCX2 isoforms, with EC_50_ values in the low nanomolar range (0.03–2.7 nM). It does not affect NCX3 activity.	Reversible; potential therapeutic applications in stroke-related pathologies.	[89,90,91]

## 4. Conclusions

Over the past decade, remarkable progress has been made in elucidating the structure–dynamic mechanisms underlying ion transport and regulation in NCX orthologs, isoforms, and splice variants. Multidisciplinary studies on archaeal (NCX_Mj), invertebrate (CALX1–2), and mammalian (NCX1–3) exchangers have clarified key facets of their transport function. The crystallographic structure of NCX_Mj provided a foundational model of the alternating-access mechanism—where ion binding drives conformational transitions between outward- and inward-facing states—yet its lack of regulatory domains limited its relevance to eukaryotic systems [79,97]

A major breakthrough has emerged with the recent cryo-EM structures of full-length human NCX1 isoforms (e.g., NCX1.1 and NCX1.3) resolved in multiple functional states: inward-activated, Ca^2+^-bound active, and SEA0400-bound inhibited forms [13]. These structures illuminate the dynamic interplay between transmembrane (TM) ion-binding core and regulatory CBD1/CBD2 domains, revealing the molecular basis of PIP_2_ activation and SEA0400 inhibition [98]. Complementary techniques, including ^19^F-NMR and HDX-MS on reconstituted systems, promise to extend these insights across additional isoforms, while CRISPR/Cas9 models enable assessment of splice variant-specific physiological roles.

Concurrently, NCX1, NCX2, and NCX3 have been implicated in maintaining intracellular Na^+^ and Ca^2+^ homeostasis in neurodegenerative pathologies such as stroke, Alzheimer’s disease, multiple sclerosis, ALS, and spinal muscular atrophy [78]. Pharmacological stimulation of specific NCX isoforms thus offers a compelling therapeutic strategy. Early drug discovery efforts are targeting isoform-specific regulation via transcriptional activation (e.g., Akt/CREB-mediated NCX1 upregulation and proteasome-stabilized NCX3) and direct ligand effects (e.g., SEA0400 and PIP_2_ modulating NCX1 conformation and function). Structural-functional characterization—especially patch-clamp and mutagenesis analysis using these new cryo-EM models—provides a robust platform for developing “drug-like” modulators stabilizing regulatable conformations.

In summary, although challenges remain in fully mapping the dynamic landscape- and tissue-specific behavior of all NCX isoforms, the availability of full-length structures, along with integrative biophysical, computational, and gene-editing tools, marks a pivotal advance. These foundations now support the rational design of isoform-selective NCX modulators that could lead to targeted therapies for a variety of neurodegenerative and cardiovascular diseases, realizing the long-anticipated clinical potential of NCX as a druggable target. Despite these promising advances, translation of NCX-targeting compounds into clinical applications remains unrealized. Several experimental inhibitors and activators, such as KB-R7943, SEA0400, Neurounina-1, and YM-244769, have demonstrated neuroprotective or antiproliferative effects [99]. In cellular and animal models, but limitations related to isoform selectivity, off-target effects, pharmacokinetics, and species-specific differences have so far prevented progression to human trials. Addressing these translational challenges will be crucial for future drug discovery efforts, emphasizing the need for highly selective, safe, and bioavailable NCX modulators validated in human-relevant models to bridge the gap between preclinical promise and clinical application.

## References

[1] Pignataro G., Sirabella R., Anzilotti S., Di Renzo G., Annunziato L. (2014). Does Na^+^/Ca^2+^ Exchanger, NCX, Represent a New Druggable Target in Stroke Intervention?. Transl. Stroke Res..

[2] Annunziato L., Pignataro G., Di Renzo G.F. (2004). Pharmacology of Brain Na^+^/Ca^2+^ Exchanger: From Molecular Biology to Therapeutic Perspectives. Pharmacol. Rev..

[3] Annunziato L. (2013). Sodium Calcium Exchange: A Growing Spectrum of Pathophysiological Implications. Proceedings of the 6th International Conference on Sodium Calcium Exchange, Lacco Ameno, Italy, 1–5 October 2011.

[4] Pannaccione A., Piccialli I., Secondo A., Ciccone R., Molinaro P., Boscia F., Annunziato L. (2020). The Na^+^/Ca^2+^ exchanger in Alzheimer’s Disease. Cell Calcium.

[5] Khananshvili D. (2014). Sodium-Calcium Exchangers (NCX): Molecular Hallmarks Underlying the Tissue-Specific and Systemic Functions. Pflugers Arch..

[6] Lytton J. (2007). Na^+^/Ca^2+^ Exchangers: Three Mammalian Gene Families Control Ca^2+^ Transport. Biochem. J..

[7] Morales A., Lachuer J., Bilbaut A., Georges B., Andrieu J.-L., Diez J., Ojeda C. (2001). Characterization of a Na^+^-Ca^2+^ Exchanger NCX1 Isoform in Bovine Fasciculata Cells of Adrenal Gland. Mol. Cell. Biochem..

[8] Sosnoski D.M., Gay C.V. (2008). NCX3 Is a Major Functional Isoform of the Sodium–Calcium Exchanger in Osteoblasts. J. Cell. Biochem..

[9] Szewczyk M.M., Davis K.A., Samson S.E., Simpson F., Rangachari P.K., Grover A.K. (2007). Ca^2+^-pumps and Na^+^-Ca^2+^-exchangers in coronary artery endothelium versus smooth muscle. J. Cell. Mol. Med..

[10] Hilgemann D.W., Collins A., Matsuoka S. (1992). Steady-State and Dynamic Properties of Cardiac Sodium-Calcium Exchange. Secondary Modulation by Cytoplasmic Calcium and ATP. J. Gen. Physiol..

[11] Hilgemann D.W., Matsuoka S., Nagel G.A., Collins A. (1992). Steady-State and Dynamic Properties of Cardiac Sodium-Calcium Exchange. Sodium-Dependent Inactivation. J. Gen. Physiol..

[12] Giladi M., Lee S.Y., Ariely Y., Teldan Y., Granit R., Strulovich R., Haitin Y., Chung K.Y., Khananshvili D. (2017). Structure-Based Dynamic Arrays in Regulatory Domains of Sodium-Calcium Exchanger (NCX) Isoforms. Sci. Rep..

[13] Xue J., Zeng W., Han Y., John S., Ottolia M., Jiang Y. (2023). Structural Mechanisms of the Human Cardiac Sodium-Calcium Exchanger NCX1. Nat. Commun..

[14] Giladi M., Fojtík L., Strauss T., Da’adoosh B., Hiller R., Man P., Khananshvili D. (2024). Structural Dynamics of Na^+^ and Ca^2+^ Interactions with Full-Size Mammalian NCX. Commun. Biol..

[15] Boyman L., Hagen B.M., Giladi M., Hiller R., Lederer W.J., Khananshvili D. (2011). Proton-Sensing Ca^2+^ Binding Domains Regulate the Cardiac Na^+^/Ca^2+^ Exchanger. J. Biol. Chem..

[16] Besserer G.M., Ottolia M., Nicoll D.A., Chaptal V., Cascio D., Philipson K.D., Abramson J. (2007). The Second Ca^2+^-Binding Domain of the Na^+^-Ca^2+^ Exchanger Is Essential for Regulation: Crystal Structures and Mutational Analysis. Proc. Natl. Acad. Sci. USA.

[17] Nicoll D.A., Sawaya M.R., Kwon S., Cascio D., Philipson K.D., Abramson J. (2006). The Crystal Structure of the Primary Ca^2+^ Sensor of the Na^+^/Ca^2+^ Exchanger Reveals a Novel Ca^2+^ Binding Motif. J. Biol. Chem..

[18] Matsuoka S., Nicoll D.A., He Z., Philipson K.D. (1997). Regulation of the Cardiac Na^+^-Ca^2+^ Exchanger by the Endogenous XIP Region. J. Gen. Physiol..

[19] Molinaro P., Pannaccione A., Sisalli M.J., Secondo A., Cuomo O., Sirabella R., Cantile M., Ciccone R., Scorziello A., di Renzo G. (2015). A New Cell-Penetrating Peptide That Blocks the Autoinhibitory XIP Domain of NCX1 and Enhances Antiporter Activity. Mol. Ther..

[20] Gök C., Fuller W. (2020). Regulation of NCX1 by Palmitoylation. Cell Calcium.

[21] Gök C., Plain F., Robertson A.D., Howie J., Baillie G.S., Fraser N.J., Fuller W. (2020). Dynamic Palmitoylation of the Sodium-Calcium Exchanger Modulates Its Structure, Affinity for Lipid-Ordered Domains, and Inhibition by XIP. Cell Rep..

[22] Gök C., Fuller W. (2024). Rise of Palmitoylation: A New Trick to Tune NCX1 Activity. Biochim. Biophys. Acta Mol. Cell Res..

[23] Scorziello A., Savoia C., Sisalli M.J., Adornetto A., Secondo A., Boscia F., Esposito A., Polishchuk E.V., Polishchuk R.S., Molinaro P. (2013). NCX3 Regulates Mitochondrial Ca^2+^ Handling through the AKAP121-Anchored Signaling Complex and Prevents Hypoxia-Induced Neuronal Death. J. Cell Sci..

[24] Sisalli M.J., Feliciello A., Della Notte S., Di Martino R., Borzacchiello D., Annunziato L., Scorziello A. (2020). Nuclear-Encoded NCX3 and AKAP121: Two Novel Modulators of Mitochondrial Calcium Efflux in Normoxic and Hypoxic Neurons. Cell Calcium.

[25] Canitano A., Papa M., Boscia F., Castaldo P., Sellitti S., Taglialatela M., Annunziato L. (2002). Brain Distribution of the Na^+^/Ca^2+^ Exchanger-Encoding Genes NCX1, NCX2, and NCX3 and Their Related Proteins in the Central Nervous System. Ann. N. Y. Acad. Sci..

[26] Secondo A., Staiano R.I., Scorziello A., Sirabella R., Boscia F., Adornetto A., Valsecchi V., Molinaro P., Canzoniero L.M.T., Di Renzo G. (2007). BHK Cells Transfected with NCX3 Are More Resistant to Hypoxia Followed by Reoxygenation than Those Transfected with NCX1 and NCX2: Possible Relationship with Mitochondrial Mem-brane Potential. Cell Calcium.

[27] Boscia F., Gala R., Pignataro G., de Bartolomeis A., Cicale M., Ambesi-Impiombato A., Di Renzo G., Annunziato L. (2006). Permanent Focal Brain Ischemia Induces Isoform-Dependent Changes in the Pattern of Na^+^/Ca^2+^ Exchanger Gene Expression in the Ischemic Core, Periinfarct Area, and Intact Brain Regions. J. Cereb. Blood Flow Metab..

[28] Pignataro G., Esposito E., Cuomo O., Sirabella R., Boscia F., Guida N., Di Renzo G., Annunziato L. (2011). The NCX3 Isoform of the Na^+^/Ca^2+^ Exchanger Contributes to Neuroprotection Elicited by Ischemic Postconditioning. J. Cereb. Blood Flow Metab..

[29] Querfurth H.W., LaFerla F.M. (2010). Alzheimer’s disease. N. Engl. J. Med..

[30] Piccialli I., Ciccone R., Secondo A., Boscia F., Tedeschi V., de Rosa V., Cepparulo P., Annunziato L., Pannaccione A. (2021). The Na^+^/Ca^2+^ Exchanger 3 Is Functionally Coupled with the Na_V_1.6 Voltage-Gated Channel and Promotes an Endoplasmic Reticulum Ca^2+^ Refilling in a Transgenic Model of Alzheimer’s Disease. Front. Pharmacol..

[31] Berridge M.J. (2010). Calcium hypothesis of Alzheimer’s disease. Pflugers Arch..

[32] De Felice F.G., Velasco P.T., Lambert M.P., Viola K., Fernandez S.J., Ferreira S.T., Klein W.L. (2007). Aβ Oligomers Induce Neuronal Oxidative Stress through an N-Methyl-D-Aspartate Receptor-Dependent Mechanism That Is Blocked by the Alzheimer Drug Memantine. J. Biol. Chem..

[33] Sokolow S., Luu S.H., Headley A.J., Hanson A.Y., Kim T., Miller C.A., Vinters H.V., Gylys K.H. (2011). High Levels of Synaptosomal Na^+^-Ca^2+^ Exchangers (NCX1, NCX2, NCX3) Co-Localized with Amyloid-Beta in Human Cerebral Cortex Affected by Alzheimer’s Disease. Cell Calcium.

[34] Natale S., Anzilotti S., Petrozziello T., Ciccone R., Serani A., Calabrese L., Severino B., Frecentese F., Secondo A., Pannaccione A. (2020). Genetic Up-Regulation or Pharmacological Activation of the Na^+^/Ca^2+^ Exchanger 1 (NCX1) Enhances Hippocampal-Dependent Contextual and Spatial Learning and Memory. Mol. Neurobiol..

[35] Boscia F., de Rosa V., Cammarota M., Secondo A., Pannaccione A., Annunziato L. (2020). The Na^+^/Ca^2+^ Exchangers in Demyelinating Diseases. Cell Calcium.

[36] Boscia F., D’Avanzo C., Pannaccione A., Secondo A., Casamassa A., Formisano L., Guida N., Annunziato L. (2012). Silencing or Knocking out the Na^+^/Ca^2+^ Exchanger-3 (NCX3) Impairs Oligodendrocyte Differentiation. Cell Death Differ..

[37] Cammarota M., de Rosa V., Pannaccione A., Secondo A., Tedeschi V., Piccialli I., Fiorino F., Severino B., Annunziato L., Boscia F. (2021). Rebound Effects of NCX3 Pharmacological Inhibition: A Novel Strategy to Accelerate Myelin Formation in Oligodendrocytes. Biomed. Pharmacother..

[38] Spillantini M.G., Crowther R.A., Jakes R., Hasegawa M., Goedert M. (1998). α-Synuclein in Filamentous Inclusions of Lewy Bodies from Parkinson’s Disease and Dementia with Lewy Bodies. Proc. Natl. Acad. Sci. USA.

[39] Bastioli G., Piccirillo S., Castaldo P., Magi S., Tozzi A., Amoroso S., Calabresi P. (2019). Selective Inhibition of Mitochondrial Sodium-Calcium Exchanger Protects Striatal Neurons from α-Synuclein plus Rotenone Induced Toxicity. Cell Death Dis..

[40] Wood-Kaczmar A., Deas E., Wood N.W., Abramov A.Y. (2013). The Role of the Mitochondrial NCX in the Mechanism of Neurodegeneration in Parkinson’s Disease. Adv. Exp. Med. Biol..

[41] Sirabella R., Sisalli M.J., Costa G., Omura K., Ianniello G., Pinna A., Morelli M., Di Renzo G.M., Annunziato L., Scorziello A. (2018). NCX1 and NCX3 as Potential Factors Contributing to Neurodegeneration and Neuroinflammation in the A53T Transgenic Mouse Model of Parkinson’s Disease. Cell Death Dis..

[42] Golovko M.Y., Barceló-Coblijn G., Castagnet P.I., Austin S., Combs C.K., Murphy E.J. (2009). The Role of α-Synuclein in Brain Lipid Metabolism: A Downstream Impact on Brain Inflammatory Response. Mol. Cell. Biochem..

[43] Appel S.H. (2006). Is ALS a Systemic Disorder? Evidence from Muscle Mitochondria. Exp. Neurol..

[44] Rosen D.R., Siddique T., Patterson D., Figlewicz D.A., Sapp P., Hentati A., Donaldson D., Goto J., O’Regan J.P., Deng H.-X. (1993). Mutations in Cu/Zn Superoxide Dismutase Gene Are Associated with Familial Amyotrophic Lateral Sclerosis. Nature.

[45] Petrozziello T., Secondo A., Tedeschi V., Esposito A., Sisalli M., Scorziello A., Di Renzo G., Annunziato L. (2017). ApoSOD1 Lacking Dismutase Activity Neuroprotects Motor Neurons Exposed to Beta-Methylamino-L-Alanine through the Ca^2+^/Akt/ERK1/2 Prosurvival Pathway. Cell Death Differ..

[46] Cozzolino M., Pesaresi M.G., Gerbino V., Grosskreutz J., Carrì M.T. (2012). Amyotrophic Lateral Sclerosis: New Insights into Underlying Molecular Mechanisms and Opportunities for Therapeutic Intervention. Antioxid. Redox Signal..

[47] Anzilotti S., Brancaccio P., Simeone G., Valsecchi V., Vinciguerra A., Secondo A., Petrozziello T., Guida N., Sirabella R., Cuomo O. (2018). Preconditioning, Induced by Sub-Toxic Dose of the Neurotoxin L-BMAA, Delays ALS Progression in Mice and Prevents Na^+^/Ca^2+^ Exchanger 3 Downregulation. Cell Death Dis..

[48] Anzilotti S., Valsecchi V., Brancaccio P., Guida N., Laudati G., Tedeschi V., Petrozziello T., Frecentese F., Magli E., Hassler B. (2021). Prolonged NCX Activation Prevents SOD1 Accumulation, Reduces Neuroinflammation, Ameliorates Motor Behavior and Prolongs Survival in a ALS Mouse Model. Neurobiol. Dis..

[49] Petrozziello T., Boscia F., Tedeschi V., Pannaccione A., de Rosa V., Corvino A., Severino B., Annunziato L., Secondo A. (2022). Na^+^/Ca^2+^ Exchanger Isoform 1 Takes Part to the Ca^2+^-Related Prosurvival Pathway of SOD1 in Primary Motor Neurons Exposed to Beta-Methylamino-l-Alanine. Cell Commun. Signal..

[50] Newton H.B. (2008). Glioblastoma Multiforme. Curr. Treat. Options Neurol..

[51] Brandalise F., Ratto D., Leone R., Olivero F., Roda E., Locatelli C.A., Grazia Bottone M., Rossi P. (2020). Deeper and Deeper on the Role of BK and Kir4.1 Channels in Glioblastoma Invasiveness: A Novel Summative Mechanism?. Front. Neurosci..

[52] Ratto D., Ferrari B., Roda E., Brandalise F., Siciliani S., De Luca F., Priori E.C., Di Iorio C., Cobelli F., Veneroni P. (2020). Squaring the Circle: A New Study of Inward and Outward-Rectifying Potassium Currents in U251 GBM Cells. Cell. Mol. Neurobiol..

[53] Turner K.L., Sontheimer H. (2014). Cl^−^ and K^+^ Channels and Their Role in Primary Brain Tumour Biology. Philos. Trans. R. Soc. Lond. B Biol. Sci..

[54] Brandalise F., Ramieri M., Pastorelli E., Priori E.C., Ratto D., Venuti M.T., Roda E., Talpo F., Rossi P. (2023). Role of Na^+^/Ca^2+^ Exchanger (NCX) in Glioblastoma Cell Migration (In Vitro). Int. J. Mol. Sci..

[55] Song M., Chen D., Yu S.P. (2014). The TRPC Channel Blocker SKF96365 Inhibits Glioblastoma Cell Growth by Enhancing Reverse Mode of the Na^+^/Ca^2+^ Exchanger and Increasing Intracellular Ca^2+^. Br. J. Pharmacol..

[56] Hu H., Wang S., Wang Y., Liu Y., Feng X., Shen Y., Zhu L., Chen H., Song M. (2019). Blockade of the Forward Na^+^/Ca ^2+^ Exchanger Suppresses the Growth of Glioblastoma Cells through Ca^2+^-mediated Cell Death. Br. J. Pharmacol..

[57] Amoroso S., Taglialatela M., Canzoniero L.M.T., Cragoe E.J., di Renzo G., Annunziato L. (1990). Possible Involvement of Ca^++^ Ions, Protein Kinase C and Na^+^-H^+^ Antiporter in Insulin-Induced Endogenous Dopamine Release from Tuberoinfundibular Neurons. Life Sci..

[58] Liu Z., Cheng Q., Ma X., Song M. (2022). Suppressing Effect of Na^+^/Ca^2+^ Exchanger (NCX) Inhibitors on the Growth of Melanoma Cells. Int. J. Mol. Sci..

[59] Rogister F., Laeckmann D., Plasman P.O., Van Eylen F., Ghyoot M., Maggetto C., Liégeois J.-F., Géczy J., Herchuelz A., Delarge J. (2001). Novel Inhibitors of the Sodium–Calcium Exchanger: Benzene Ring Analogues of N-Guanidino Substituted Amiloride Derivatives. Eur. J. Med. Chem..

[60] Secondo A., Pannaccione A., Molinaro P., Ambrosino P., Lippiello P., Esposito A., Cantile M., Khatri P.R., Melisi D., Di Renzo G. (2009). Molecular Pharmacology of the Amiloride Analog 3-Amino-6-Chloro-5-[(4-Chloro-Benzyl)Amino]-N-[[(2,4-Dimethylbenzyl)-Amino]Iminomethyl]-Pyrazinecarboxamide (CB-DMB) as a Pan Inhibitor of the Na^+^-Ca^2+^ Exchanger Isoforms NCX1, NCX2, and NCX3 in Stably Transfected Cells. J. Pharmacol. Exp. Ther..

[61] Shigekawa M., Iwamoto T. (2001). Cardiac Na^+^-Ca^2+^ Exchange. Circ. Res..

[62] Watano T., Kimura J., Morita T., Nakanishi H. (1996). A Novel Antagonist, No. 7943, of the Na^+^/Ca^2+^ Exchange Current in Guinea-Pig Cardiac Ventricular Cells. Br. J. Pharmacol..

[63] Iwamoto T., Watano T., Shigekawa M. (1996). A Novel Isothiourea Derivative Selectively Inhibits the Reverse Mode of Na^+^/Ca^2+^ Exchange in Cells Expressing NCX1. J. Biol. Chem..

[64] Amran M.S., Homma N., Hashimoto K. (2003). Pharmacology of KB-R7943: A Na^+^-Ca^2+^ Exchange Inhibitor. Cardiovasc. Drug Rev..

[65] Sobolevsky A.I., Khodorov B.I. (1999). Blockade of NMDA Channels in Acutely Isolated Rat Hippocampal Neurons by the Na^+^/Ca^2+^ Exchange Inhibitor KB-R7943. Neuropharmacology.

[66] Pintado A.J., Herrero C.J., García A.G., Montiel C. (2000). The Novel Na^+^/Ca^2+^ Exchange Inhibitor KB-R7943 Also Blocks Native and Expressed Neuronal Nicotinic Receptors. Br. J. Pharmacol..

[67] MacDonald A.C., Howlett S.E. (2008). Differential Effects of the Sodium Calcium Exchange Inhibitor, KB-R7943, on Ischemia and Reperfusion Injury in Isolated Guinea Pig Ventricular Myocytes. Eur. J. Pharmacol..

[68] Andreeva-Gateva P., Hristov M., Strokova-Stoilova M., Ivanova N., Sabit Z., Surcheva S., Beliakov M., Karakashev G., Sukhov I., Belinskaya D. (2024). Therapeutic potential of orally applied KB-R7943 in streptozotocin-induced neuropathy in rats. Heliyon.

[69] Matsuda T., Arakawa N., Takuma K., Kishida Y., Kawasaki Y., Sakaue M., Takahashi K., Takahashi T., Suzuki T., Ota T. (2001). SEA0400, a novel and selective inhibitor of the Na^+^-Ca^2+^ exchanger, attenuates reperfusion injury in the in vitro and in vivo cerebral ischemic models. J. Pharmacol. Exp. Ther..

[70] Tanaka H., Nishimaru K., Aikawa T., Hirayama W., Tanaka Y., Shigenobu K. (2002). Effect of SEA0400, a Novel In-hibitor of Sodium-calcium Exchanger, on Myocardial Ionic Currents. Br. J. Pharmacol..

[71] Matsuda T., Koyama Y., Baba A. (2005). Functional Proteins Involved in Regulation of Intracellular Ca^2+^ for Drug Development: Pharmacology of SEA0400, a Specific Inhibitor of the Na^+^-Ca^2+^ Exchanger. J. Pharmacol. Sci..

[72] Kita S., Iwamoto T. (2007). Inhibitory Mechanism of SN-6, A Novel Benzyloxyphenyl Na^+^/Ca^2+^ Exchange Inhibitor. Ann. N. Y. Acad. Sci..

[73] Iwamoto T., Inoue Y., Ito K., Sakaue T., Kita S., Katsuragi T. (2004). The Exchanger Inhibitory Peptide Region-Dependent Inhibition of Na^+^ /Ca^2+^ Exchange by SN-6 [2-[4-(4-Nitrobenzyloxy)Benzyl]Thiazolidine-4-Carboxylic Acid Ethyl Ester], a Novel Benzyloxyphenyl Derivative. Mol. Pharmacol..

[74] Kuramochi T., Kakefuda A., Yamada H., Ogiyama T., Taguchi T., Sakamoto S. (2005). Synthesis and Structure–Activity Relationships of Benzyloxyphenyl Derivatives as a Novel Class of NCX Inhibitors: Effects on Heart Failure. Bioorg. Med. Chem..

[75] Kuramochi T., Kakefuda A., Sato I., Tsukamoto I., Taguchi T., Sakamoto S. (2005). Synthesis and Structure–Activity Relationships of 6-{4-[(3-Fluorobenzyl)oxy]Phenoxy}nicotinamide Derivatives as a Novel Class of NCX Inhibitors: A QSAR Study. Bioorg. Med. Chem..

[76] Iwamoto T., Kita S. (2006). YM-244769, a Novel Na^+^/Ca^2+^ Exchange Inhibitor That Preferentially Inhibits NCX3, Effi-ciently Protects against Hypoxia/Reoxygenation-Induced SH-SY5Y Neuronal Cell Damage. Mol. Pharmacol..

[77] Secondo A., Pignataro G., Ambrosino P., Pannaccione A., Molinaro P., Boscia F., Cantile M., Cuomo O., Esposito A., Sisalli M.J. (2015). Pharmacological Characterization of the Newly Synthesized 5-Amino-N-Butyl-2-(4-Ethoxyphenoxy)-Benzamide Hydrochloride (BED) as a Potent NCX3 Inhibitor That Worsens Anoxic Injury in Cortical Neurons, Organotypic Hippocampal Cultures, and Ischemic Brain. ACS Chem. Neurosci..

[78] Annunziato L., Secondo A., Pignataro G., Scorziello A., Molinaro P. (2020). New Perspectives for Selective NCX Activators in Neurodegenerative Diseases. Cell Calcium.

[79] Dong Y., Yu Z., Li Y., Huang B., Bai Q., Gao Y., Chen Q., Li N., He L., Zhao Y. (2024). Structural Insight into the Allosteric Inhibition of Human Sodium-Calcium Exchanger NCX1 by XIP and SEA0400. EMBO J..

[80] Hasegawa H., Muraoka M., Matsui K., Kojima A. (2003). Discovery of a Novel Potent Na^+^/Ca^2+^ Exchanger Inhibitor: Design, Synthesis and Structure–Activity Relationships of 3,4-Dihydro-2(1 H)-Quinazolinone Derivatives. Bioorg. Med. Chem. Lett..

[81] Hasegawa H., Muraoka M., Ohmori M., Matsui K., Kojima A. (2005). A Novel Class of Sodium/Calcium Exchanger Inhibitor: Design, Synthesis, and Structure–Activity Relationships of 3,4-Dihydro-2(1H)-Quinazolinone Derivatives. Bioorg. Med. Chem..

[82] Otsomaa L., Levijoki J., Wohlfahrt G., Chapman H., Koivisto A.P., Syrjänen K., Koskelainen T., Peltokorpi S.E., Finckenberg P., Heikkilä A. (2020). Discovery and Characterization of ORM-11372, a Novel Inhibitor of the Sodium-Calcium Exchanger with Positive Inotropic Activity. Br. J. Pharmacol..

[83] Giacomello M., Drago I., Pizzo P., Pozzan T. (2007). Mitochondrial Ca^2+^ as a Key Regulator of Cell Life and Death. Cell Death Differ..

[84] Szabadkai G., Simoni A.M., Bianchi K., De Stefani D., Leo S., Wieckowski M.R., Rizzuto R. (2006). Mitochondrial Dynamics and Ca^2+^ Signaling. Biochim. Biophys. Acta.

[85] Castaldo P., Cataldi M., Magi S., Lariccia V., Arcangeli S., Amoroso S. (2009). Role of the Mitochondrial Sodium/Calcium Exchanger in Neuronal Physiology and in the Pathogenesis of Neurological Diseases. Prog. Neurobiol..

[86] Quan X., Nguyen T.T., Choi S.-K., Xu S., Das R., Cha S.-K., Kim N., Han J., Wiederkehr A., Wollheim C.B. (2015). Essential Role of Mitochondrial Ca^2+^ Uniporter in the Generation of Mitochondrial PH Gradient and Metabolism-Secretion Coupling in Insulin-Releasing Cells. J. Biol. Chem..

[87] Martínez-Sanz F.J., Lajarín-Cuesta R., Moreno-Ortega A.J., González-Lafuente L., Fernández-Morales J.C., López-Arribas R., Cano-Abad M.F., De Los Ríos C. (2015). Benzothiazepine CGP37157 Analogues Exert Cytoprotection in Various in Vitro Models of Neurodegeneration. ACS Chem. Neurosci..

[88] González-Lafuente L., Egea J., León R., Martínez-Sanz F.J., Monjas L., Perez C., Merino C., García-De Diego A.M., Rodríguez-Franco M.I., García A.G. (2012). Benzothiazepine CGP37157 and Its Isosteric 2′-Methyl Analogue Provide Neuroprotection and Block Cell Calcium Entry. ACS Chem. Neurosci..

[89] Molinaro P., Cantile M., Cuomo O., Secondo A., Pannaccione A., Ambrosino P., Pignataro G., Fiorino F., Severino B., Gatta E. (2013). Neurounina-1, a Novel Compound That Increases Na^+^ /Ca^2+^ Exchanger Activity, Effectively Protects against Stroke Damage. Mol. Pharmacol..

[90] Severino B., Corvino A., Fiorino F., Frecentese F., Perissutti E., Caliendo G., Santagada V., Magli E., Molinaro P., Pignataro G. (2019). Development, Validation of LC-MS/MS Method and Determination of Pharmacokinetic Parameters of the Stroke Neuroprotectant Neurounina-1 in Beagle Dog Plasma After Intravenous Administration. Front. Pharmacol..

[91] Magli E., Fattorusso C., Persico M., Corvino A., Esposito G., Fiorino F., Luciano P., Perissutti E., Santagada V., Severino B. (2021). New Insights into the Structure–Activity Relationship and Neuroprotective Profile of Benzodiazepinone Derivatives of Neurounina-1 as Modulators of the Na^+^/Ca^2+^ Exchanger Isoforms. J. Med. Chem..

[92] Iwamoto T. (2004). Forefront of Na^+^/Ca^2+^ exchanger studies: Molecular pharmacology of Na^+^/Ca^2+^ exchange inhibitors. J. Pharmacol. Sci..

[93] Niu C.F., Watanabe Y., Ono K., Iwamoto T., Yamashita K., Satoh H., Urushida T., Hayashi H., Kimura J. (2007). Characterization of SN-6, a novel Na^+^/Ca^2+^ exchange inhibitor in guinea pig cardiac ventricular myocytes. Eur. J. Pharmacol..

[94] Jost N., Nagy N., Corici C., Kohajda Z., Horváth A., Acsai K., Biliczki P., Levijoki J., Pollesello P., Koskelainen T. (2013). ORM-10103, a novel specific inhibitor of the Na^+^/Ca^2+^ exchanger, decreases early and delayed afterdepolarizations in the canine heart. Br. J. Pharmacol..

[95] Kohajda Z., Farkas-Morvay N., Jost N., Nagy N., Geramipour A., Horváth A., Varga R.S., Hornyik T., Corici C., Acsai K. (2016). The Effect of a Novel Highly Selective Inhibitor of the Sodium/Calcium Exchanger (NCX) on Cardiac Arrhythmias in In Vitro and In Vivo Experiments. PLoS ONE.

[96] Cox D.A., Conforti L., Sperelakis N., Matlib M.A. (1993). Selectivity of inhibition of Na^+^-Ca^2+^ exchange of heart mitochondria by benzothiazepine CGP-37157. J. Cardiovasc. Pharmacol..

[97] Giladi M., Tal I., Khananshvili D. (2016). Structural Features of Ion Transport and Allosteric Regulation in Sodium-Calcium Exchanger (NCX) Proteins. Front. Physiol..

[98] Xue J., Zeng W., John S., Attiq N., Ottolia M., Jiang Y. (2025). Structural Mechanisms of PIP2 Activation and SEA0400 Inhibition in Human Cardiac Sodium-Calcium Exchanger NCX1. eLife.

[99] Galvankova K., Rezuchova I., Klena L., Grman M., Gazova S., Liskova V., Kozovska Z., Roller L., Babula P., Krizanova O. (2025). Role of the sodium/calcium exchanger type 3 in cancer cells. Eur. J. Cell Biol..

